# Age-related differences in GABAergic synapses across the central inferior colliculus in the Fischer Brown Norway rat

**DOI:** 10.1016/j.neurobiolaging.2025.06.002

**Published:** 2025-06-06

**Authors:** Alexa Wawrzyniak, Justine Busby, Alice Dalo, Syllissa Duncan, Laila S. Almassri, Dakota Z. Smallridge, Andrew P. Ohl, Amir M. Mafi, Nick J. Tokar, Jesse W. Young, Jeffrey G. Mellott

**Affiliations:** a Department of Anatomy and Neurobiology, Northeast Ohio Medical University, Rootstown, OH, USA; b University Hospitals Hearing Research Center, Northeast Ohio Medical University, Rootstown, OH, USA; c The Ohio State College of Medicine, Columbus, OH, USA

**Keywords:** central inferior colliculus, GABA, synapse, aging, ultrastructure

## Abstract

Presbycusis, one of the most widespread disorders, is in part associated with the loss of temporal precision within the central auditory system. A contributor to the dysfunctional temporal precision during aging is the substantial downregulation of GABA in the central inferior colliculus (ICc), the hub of ascending and descending inputs of the auditory midbrain. However, how GABAergic inputs across the tonotopic axis of the ICc change with age has not been well explored. We sought to determine age-related changes to GABAergic synapses in the lemniscal ICc, and if changes are uniform across the ICc axis. Using immuno-electron microscopy across four age groups of Fisher Brown Norway rats, a model that acquires low frequency presbycusis, our results demonstrate several nonuniform ultrastructural changes to GABAergic synapses in the ICc. There was a significant (~29–33 %) downregulation of GABAergic synapses in the high and middle frequency regions of old rats, but a loss (~22 %) in the old low frequency region was not as robust and did not reach statistical significance. Interestingly, in the high and middle frequency regions, GABAergic presynaptic area increased with age, while there was an ultimately decline in the old low frequency region. Also unique to the high and middle frequencies was the increasing proportion of GABAergic synapses onto larger GABAergic dendrites. These changes demonstrate that aging differentially affects the GABAergic ultrastructure of the ICc tonotopic axis.

## 1. Introduction

Age-related hearing loss (presbycusis) affects the majority of the older adult population and can be characterized by a variety of peripheral and central processing deficits that ultimately lead to social isolation, perceptual deficits, cognitive decline, and a poor quality of life (Goman and Lin, 2016; Caspary and Llano, 2018; Helfer and Bartlett, 2020). Specifically, communication is impaired due to poor speech comprehension, especially in noisy social settings, as temporal processing gradually degrades over decades of life (see reviews Frisina, 2005, 2009; Liberman, 2017; Helfer and Bartlett, 2020). A common explanation is that the central auditory system will utilize mechanisms to adjust neural gain as excitation from the cochlea degrades during aging (Tremblay et al., 2004; Caspary et al., 2008; Auerbach et al., 2014; Gold and Bajo, 2014; Parthasarathy et al., 2019a,b; Harris, 2020; Harris et al., 2022; Rumschlag et al., 2022; Dias et al., 2024). One of the best understood reasonings to this central gain mechanism is that the central auditory system, especially in the inferior colliculus (IC), downregulates γ-amino butyric acid (GABA) neurotransmission to preserve a homeostatic balance between excitation and inhibition (Richardson et al., 2012; Caspary et al., 2008; Caspary and Llano, 2018; Syka, 2020). However, the loss of GABA in the IC eventually leads to an increase in central gain that may contribute to potential deficits such as poor temporal processing in noise, hyperacusis, and tinnitus (Walton et al., 2002; Caspary et al., 2008; Walton, 2010; Parthasarathy and Bartlett, 2011, Pollak et al., 2011; Cai et al., 2014 Auerbach et al., 2014, 2019)

The IC is the largest nucleus in the auditory midbrain and serves as the hub for numerous ascending and descending auditory (as well as somatosensory and visual) inputs (Oliver et al., 1997; Cant, 2005; Oliver, 2005; Schofield, 2005; Ito and Malmierca, 2018). Studies of the IC consider at least three subdivisions: central IC (ICc), lateral cortex of the IC (IClc), and dorsal cortex of the IC (ICd). Roughly 20–25 % of the neurons in each subdivision are GABAergic, with the remaining 75–80 % expressing glutamate (Oliver et al., 1994; Merchán et al., 2005; Ito et al., 2011; Mellott et al., 2014a,b; Beebe et al., 2016). GABA is essential in the IC as it shapes and encodes many spectral and temporal responses to complex stimuli (e.g. speech; Faingold et al., 1989; Le Beau et al., 1996; Frisina, 2001; Pollak et al., 2011; Ono et al., 2017). The IC processes intrinsic, commissural and extrinsic sources of GABA (González-Hernández et al., 1996). There is a large volume of literature that demonstrates age-related changes to GABAergic neurotransmission in the IC, however little is known about how aging affects the ultrastructure of GABAergic synapses (Milbrandt et al., 1996, 1997; Caspary et al., 2008; Syka, 2020).

Helfert et al., (1999) examined GABAergic and excitatory synapses in the mid-frequency region of the tonotopically organized ICc and found a balanced downregulated (~25–30 %) with age of both synaptic types. Broadly, the morphologies of ICc neurons are classified by their dendritic branching occurring in parallel (disc-shaped/flat) or traveling across (stellate/less-flat) the laminae (Oliver and Morest, 1984; Malmierca et al., 1995; Wallace et al., 2021). However, there may be significant variations across species as the rat IC appears to have a discontinuous stepwise tonotopic gradient and fewer/no intralaminar compartments at the lower frequencies, thus there may be fewer stellate cells at lower frequencies (Malmierca et al., 1993, 1995, 2005, 2008). In general, six distinct firing patterns were recognized in the ICc, however firing pattern and the morphology of the IC cells is not as tightly coupled as it is in the lower brainstem (Sivaramakrishnan and Oliver, 2006). Recently, in the guinea pig, basket cells were recognized as a third morphological type of ICc neuron (Wallace et al., 2021). As compared to disc-shaped and stellate cells, these basket cells had unique temporal responses as they have chopper responses with 7–9 ms latencies (Wallace et al., 2021). Recent work has shed a strong light on correlating the physiology and anatomy of IC cells based on neuropeptide expression and cell size in respect to neurochemical profiles (Goyer et al., 2019; Ito, 2020; Silveira et al., 2020, 2023; Kreeger et al., 2021). Regarding aging, whether synaptic loss/changes are uniform across the dorsolateral-ventromedial tonotopic axis, and when these losses occur, is important to understand as presbycusis is often specific to either high or low frequencies (Walton et al., 1998; Cai et al., 2018; Parthasarathy and Kujawa, 2018; Koehler et al., 2023).

In the current study we examine age-related changes to the ultrastructure of GABAergic synapses in the high, middle, and low frequency regions of the ICc. As in our recent report, we examined three tissue blocks taken across the ventromedial-dorsolateral ICc axis (Mellott et al., 2024). We used immuno-transmission electron microscopy to analyze GABAergic ICc synapses of Fischer Brown Norway (FBN) rats across four age groups (3–4 months “young”; 19–20 months “early middle-age”; 24 months “late middle-age”; and 28–29 months “old”). It is important to note that the FBN rat acquires low frequency presbycusis (Keithley et al., 1992; Cai et al., 2018). Regardless of aging, GABAergic synaptic density, terminal area, active zone length, and vesicle pools were nearly identical across the three frequency regions at a young age. We found many uniform and nonuniform ultrastructural changes and correlations across the dorsolateral-ventromedial axis of the ICc. Of note, a significant GABAergic synaptic loss occurred in the high and middle frequency regions. While the density of GABAergic synapses was lower at old age than at young in the low frequency region, the loss was not as robust and did not reach significance. Also, the average area of GABAergic terminals in the high and middle frequency region increased with age, reflecting that smaller terminals were less common, while terminal area ultimately decreased at old age in the low frequency region. Collectively it appears that aging nonuniformly affects the ultrastructure of the ICc tonotopic axis.

## 2. Materials and methods

### 2.1. Animals

All procedures were conducted in accordance with the Northeast Ohio Medical University Institutional Animal Care and Use Committee and NIH guidelines. Results are described from 20 male Fischer Brown Norway (FBN) rats (National Institute of Aging; Bethesda, MD, USA; RRID:SCR_007317) across four age groups (5 animals per age group): 3–4 months “young”; 19–20 months “early middle-age”; 24 months “late middle-age”; and 28–29 months “old” (Tables, 1, 2, & 3). As efforts were made to minimize the number of animals and their suffering, seventeen of the twenty tissue blocks we report on were also used in our recent report on the ultrastructure of dense core vesicles (Mellott et al., 2024).

### 2.2. Perfusion and sectioning

Animals were deeply anesthetized with isoflurane and perfused transcardially with Tyrode’s solution, followed by 250 ml of 2 % glutaraldehyde and 2 % paraformaldehyde (with one exception; case R79, Blocks 93,94, and 95 [Tables 1,2&3] was perfused and stored with 3 % glutaraldehyde and 1 % paraformaldehyde) in 0.1 M phosphate buffer at a pH 7.4. After the brain was removed it was stored at 4°C in the same fixative it was perfused with in 0.1 M phosphate buffer. The brain was prepared the next morning by removing the cerebellum and cortex and blocking the remaining tissue with transverse cuts posterior to the cochlear nucleus and anterior to the thalamus. The tissue was then cut into 50 μm thick transverse sections with a Vibratome (VT1000S, Leica Microsystems, Buffalo Grove, IL, USA). The tissue was collected in six series. Series were processed as described below or stored in freezing buffer for future processing.

### 2.3. Tissue processing for EM

**Fig. 1.**
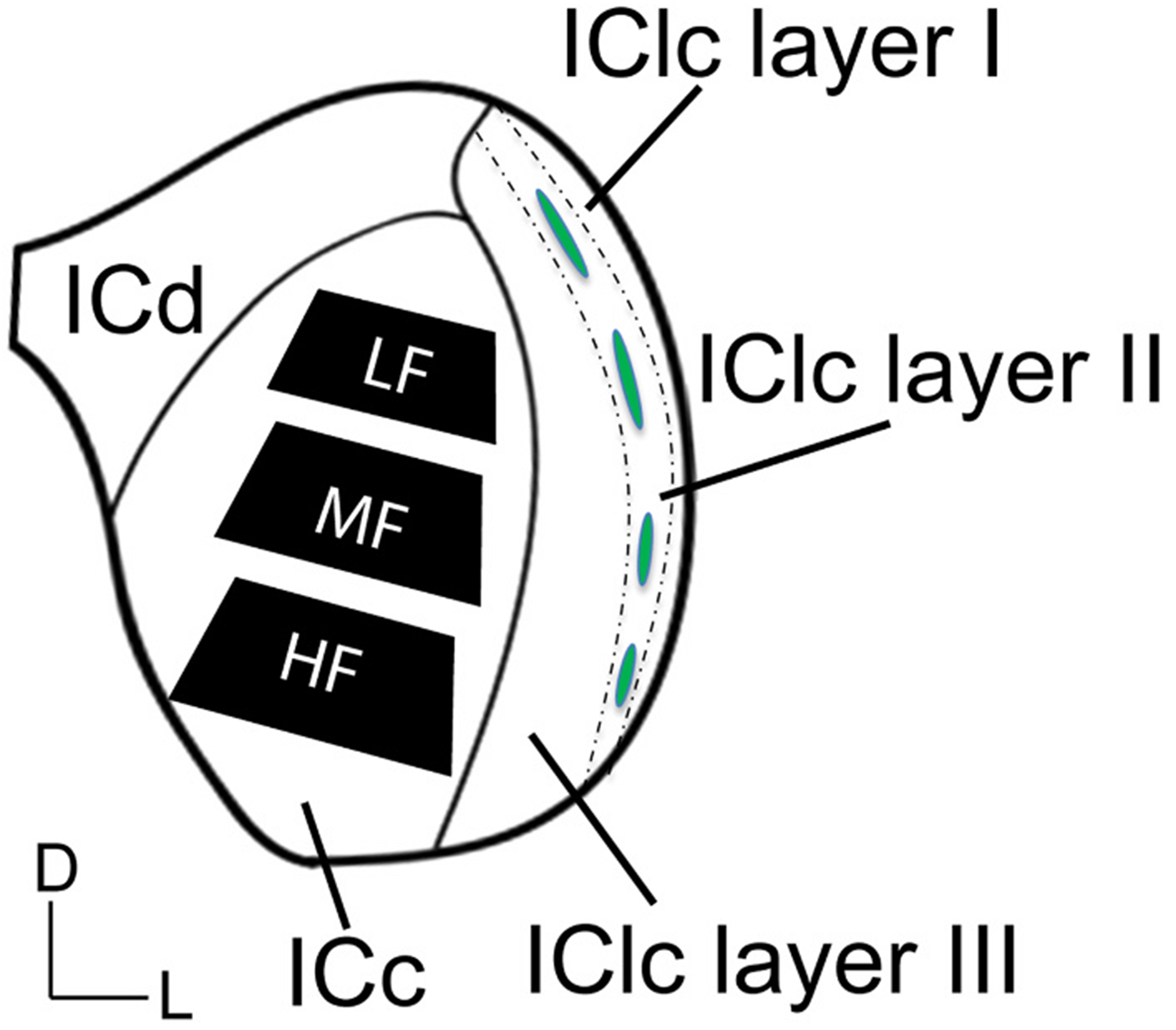
Schematic illustrating where tissue (black trapezoids) were extracted from the ICc in each case ICc across the ventromedial-dorsolateral axis. Dashed lines demonstrate the approximate borders between the three layers of the lateral cortex of the inferior colliculus (IClc). Green ovals indicate the approximate locations of the GABAergic modules that are often found and define anatomical features of the second IClc layer. D = dorsal; l = lateral; HF = high frequency region; ICc = central inferior colliculus; ICd = dorsal cortex of the IC; IClc layer I = the first layer of the lateral cortex of the IC; IClc layer II = the second layer of the IClc; IClc layer III = the third layer of the IClc; LF = low frequency region; MF = middle frequency region.

Processing for immunogold EM is identical to our previous IC studies (Mafi et al., 2022; Mellott et al., 2024). Briefly, tissue was post-fixed in 1 % osmium tetroxide for 30 min, dehydrated in a series of alcohols (50 %, 70 %, 95 %, 100 % and 2x propylene oxide), embedded in Durcupan resin (Sigma-Aldrich; Millipore Sigma, Burlington, MA, USA) and flat-mounted between sheets of Aclar Embedding Film (Ted Pella, Inc, Redding, CA, USA) at 60 degrees Celsius for 48–72 h. Mid-rostrocaudal IC sections (between interaural levels 0.24 mm – 0.36 mm; Paxinoss and Watson, 1998) were examined with brightfield stereomicroscopy. Trapezoidal blocks, with a 0.75 mm base and 0.5–0.6 mm height, were extracted across the ventromedial-dorsolateral axis of the ICc (Fig. 1). Three “blocks” of tissue were taken from each animal processed in the study. These tissue blocks were created from our recent study on dense core vesicles (Mellott et al., 2024). The ventromedial-dorsolateral axis of the rat ICc, after fixation, is approximately 2 mm. To better avoid our dorsolateral-most block including the lateral cortex of the IC (IClc) or dorsal cortex of the IC (ICd), the total length of tissue (measured with a micrometer) taken across the axis was ~1.8 mm (a 1.8 mm strip of tissue divided into thirds resulted in three 0.6 mm height blocks) to provide a 0.2 mm buffer. Initial borders of the ICc were delineated according to the rat anatomical atlas of the brain (Paxinoss and Watson, 1998). Osmium fixation revealed the conspicuous lateral lemniscal fibers that course between the ICc and the IClc. Our libraries of decarboxylase (GAD) immunoreactivity in EM prepared tissue, adjacent sections reacted for Nissl, cytochrome oxidase (CO), and our experience with EM in the IC further guided our block trimming to best ensure tissue was from ICc and that our dorsolateral “low frequency” block did not contain tissue from non-lemniscal IC (Nakamoto et al., 2013; Mellott et al., 2014c; Mafi et al., 2022; Mellott et al., 2024). In regard to ICd, there may not be a sharp anatomical or functional border with the ICc, and different histochemical approaches reveal differences across species and within the same species (Faye-Lund and Osen, 1985; Oliver, 2005; Loftus et al., 2008). Thus, we use the above approaches to give us the best picture of the border between lemniscal and non-lemniscal IC through osmium reacted axonal patterns, density of cell bodies, and CO staining mapped onto the parameters established in Paxinoss and Watson (1998) and our libraries of IC tissue labeled with a variety of immunostains. While we cannot guarantee that our dorsolateral block did not include some non-lemniscal IC, we believe that our process combined with providing a slight buffer of 0.2 mm when trimming near the ICc/ICd/IClc borders alleviates most concerns that we are including non-lemniscal IC while not excluding a large portion of the lowest ICc frequencies.

Tissue blocks were glued to a cylindric resin base with cyanoacrylate (Krazy Glue, Columbus, OH, USA). We refer to the ventromedial-most ICc block as representing the high frequency region, the middle-most block representing the middle frequency region, and dorsolateral-most block representing lower frequencies (Fig. 1). Although we did not employ electrophysiological techniques to record from the ICc, we adopt this naming convention for the sake of conciseness, brevity, and convention as the tonotopic axis and its orientation in the ICc is well established (Mellott et al., 2024).

Ultrathin sections (50 nm) were taken with an ultramicrotome (UC6 Ultramicrotome, Leica Microsystems, Buffalo Grove, IL, USA). For each tissue block, every twelfth section was collected onto a 200 or 300-mesh Formvar coated nickel mesh grid (Electron Microscopy Science, Hatfield, PA, USA) to better ensure a singular synapse was not analyzed twice. A total of eight grids, each with a single ICc ultrathin section. Briefly, (see: Nakamoto et al., 2013; Mellott et al., 2014c), sections were dried for three hours and were then placed overnight into anti-GABA antibody (rabbit anti-GABA, Sigma, St. Louis, MO) diluted 1:500 in 0.05 M Tris-buffered saline with 0.1 % Triton X-100, pH 7.6 (TBST). The next day the sections are washed in TBST pH 7.6, then washed in TBST pH 8.2, and placed into a secondary antibody conjugated to 15 nm gold particles (goat anti-rabbit, diluted 1:25 in TBST pH 8.2; Ted Pella Inc., Redding, CA). Lastly, sections were washed in TBST pH 7.6, washed in Nanopure water, stained with uranyl acetate (2 % aqueous) and Reynold’s lead citrate (Reynoldss, 1963), and airdried.

### 2.4. EM imaging

Sixty blocks of tissue from twenty male FBN rats with superior ultrastructure were chosen. A 5-point scale was used to grade the intactness and quality of ultrastructure. Only tissue with a score of 4 or 5 was quantified. Our 5-point scale reflects a combination of successful fixation, immunogold processing and absence of electron dense artifacts. Scores of 4&5 yield clear ultrastructure with easily identifiable GABAergic profiles that are readily resolved. Tissue scored as a 3 yields ultrastructure that can be qualitatively analyzed; however membrane intactness is not preserved such that quantitative data can be consistently obtained. Tissue scored as a 1 or 2 has severe defects in the pre- and postsynaptic membranes resulting in uninterpretable synaptic profiles and were not used. Ultrastructure of the ICc was imaged with a transmission electron microscope (JEM-1400Plus, JEOL, Peabody, MA, USA) at an accelerating voltage of 80 kV and at a magnification of 50,000. Based on experience, a magnification of 50,000 ensures that symmetric and GABAergic synapses in the inferior colliculus are discernable. Tissue was digitally imaged and rendered with an Orius 100 keV or Rio9 side mount camera (Gatan, Pleasanton, CA, USA). Images of ultrastructure were taken with Gatan Microscopy Suite Software (GMS3, Gatan, Pleasanton, CA, USA) integrated and calibrated with SerialEM Tomography software (Mastronarde, 2003). SerialEM is a gold standard for analytical applications in biological TEM and facilitated efficient imaging, analysis, and data recording. For each tissue block, we collected 400 μm^2^ (22,214 x 22,214 pixels) montages across 8 grids for a total of 3200 μm^2^. All attempts were made such that each montage was collected from the center of each ultrathin section. Adobe Photoshop (Adobe Systems, Inc., San Jose, CA, USA) was used to add scale bars, crop images, adjust intensity levels and colorize monochrome images.

### 2.5. Analysis of GABAergic profiles

GABAergic synapses were identified by presynaptic terminals immunopositive for gold, collections of presynaptic vesicles (often pleomorphic), and terminal symmetry (pre- and postsynaptic membranes were of similar thickness] (Rockel and Jones, 1973; Jones and Rockel, 1973; Peters et al., 1991; Paloff and Usunoff, 1992; Helfert et al., 1999; Nakamoto et al., 2013, Mafi et al., 2022, Mellott et al., 2024). Qualitatively, GABAergic profiles were readily distinguished from GABA-negative profiles by an accumulation of gold particles as compared to background (Nakamoto et al., 2013; Mellott et al., 2014b, Mafi et al., 2022; Mellott et al., 2024). In line with our previous studies on the aging ultrastructure of the IC, we found that GABAergic profiles typically had at least 3–6 times more gold particles than boutons with GABA-negative “excitatory-like” attributes (round vesicles; asymmetric PSD, Mafi et al., 2022; Mellott et al., 2024). Aggregates of two or more gold particles were quantified as a single particle and gold particles found clumped together on mitochondria were left out of quantification as this is indicative of non-specific binding. Most symmetric synapses were formed by GABAergic terminals; however, a small percentage (~8 %) of terminals forming symmetric synapses were GABA-negative and presumed to be glycinergic and were not included in the current study. Lastly, while rare (<4 %), some GABAergic terminals at 24 months and 28–29 months with pleomorphic vesicles formed synapses with large postsynaptic densities that appeared asymmetric. For the sake of quantification these synapses were considered GABAergic based on the positive immunoreactivity. Postsynaptic targets (dendrites, spines, boutons, and somata) were also identified as GABAergic or GABA-negative.

### 2.6. Data analysis

We examined 48,000 μm^2^ of ICc tissue across five 3–4-month-old tissue blocks, 48,000 μm^2^ of ICc across five 19–20-month-old tissue blocks, 48,000 μm^2^ of ICc across five 24-month-old tissue blocks, and 48,000 μm^2^ of ICc tissue across five blocks of 28–29-month-old tissue blocks (Tables 1,2&3). Each presynaptic GABAergic terminal and postsynaptic target was analyzed manually with ImageJ (Schneider et al., 2012). We collected several ultrastructural details for each synapse: 1) presynaptic area (we assigned presynaptic areas of >1.0 μm^2^ as “large” and those with areas <0.6 μm^2^ as “small”), 2) active zone length, 3) the number of total vesicles in the terminal and the number of those vesicles at the active zone (releasing pool), and 4) the number and ultrastructural grade of each presynaptic mitochondria (Coughlin et al., 2015; Smith et al., 2016; Mafi et al., 2022). We recorded the postsynaptic target of each synapse as either a GABAergic or GABA-negative soma, large dendrite, medium dendrite, small dendrite or spine. For the sake of consistency across EM studies on the aging IC, we used dendritic sizes (>1.5 μm-large; 0.5–1.5 μm-medium; <0.5 μm-small) established by Helfert et al., (1999). As detailed in Mafi et al., (2022) it is sometimes difficult to distinguish spines from small dendrites in the IC as 1) there is overlap in diameter ranges, 2) spine necks are not consistently observed at 50 nm thick sections, and 3) spine apparatus is only present in a subset of spines.

Variation in synaptic density, presynaptic area, the active zone size, number of vesicles, presynaptic mitochondria and postsynaptic targets according to age group and frequency region, were analyzed using linear mixed-effects models. Mixed-effects models allow for a hybrid of repeated measures analysis (i.e., “within-subject” variables), Model I ANOVA fixed factor analysis (i.e., “between-subject” variables), and Model II ANOVA random factor analysis (i.e., variance components), in the same gestalt statistical test. In this study, age group was specified as a between-subjects fixed factor across individual rats, individual ICc region and ultrastructural profile were specified as within-subjects fixed factors within individual rats, and individual rat number was specified as a random factor. Measures of presynaptic area and active zone size were natural-log transformed prior to model-fitting to improve normality. Models for continuous measures (i.e., presynaptic area, active zone size, and postsynaptic target diameter) and ordinal measures (i.e., mitochondrial scores) were fit assuming a gaussian error distribution (R function lmer), whereas models for count data (e.g., synaptic density, number of versicles, and presynaptic mitochondria) were fit assuming a Poisson error distribution (R function glmer). P-values for pairwise post hoc tests of differences between consecutive age groups (i.e., 3–4 months *versus* 19–20 months, 19–20 months *versus* 24 months, 24 months *versus* 28–29 months) were adjusted using the False Discovery Rate procedure (Benjamini and Hochberg, 1995), a method that simultaneously limits experiment-wise alpha inflation and minimizes the correlated loss of statistical power. All statistical tests were performed in R (version 4.4.3 for Mac OS X; R Core Team, 2025), supplemented by the add-on packages *lme4* (Bates et al., 2015), *lmerTest* (Kuznetsova et al., 2017), *emmeans* (Lenth, 2024), and *tidyverse* (Wickham et al., 2019).

## 3. Results

We examined GABAergic synapse ultrastructure in four age groups (3–4 months, 19–20 months, 24 months, and 28–29 months) in the central inferior colliculus (ICc). We quantified 5565 GABAergic synapses across 192,000 μm^2^ of tissue (Tables 1,2,3). Overall, we found that the density of GABAergic synapses at 3–4 months did not differ along the ventromedial-dorsolateral axis of the ICc, however the density steadily declined from middle to old age (compare Tables 1,2,3). We first present the changes to GABAergic synaptic density. We then present data regarding the changes to presynaptic elements (terminal size, post-synaptic targets, active zone size, vesicles pools, and mitochondria). Table 4 shows the correlation coefficients and p values of the means of these quantified elements across ages. Lastly, we describe the frequency of which GABAergic synapses form onto GABAergic and GABA-negative somata, dendrites and spines.

### 3.1. GABAergic synaptic loss

**Fig. 2.**
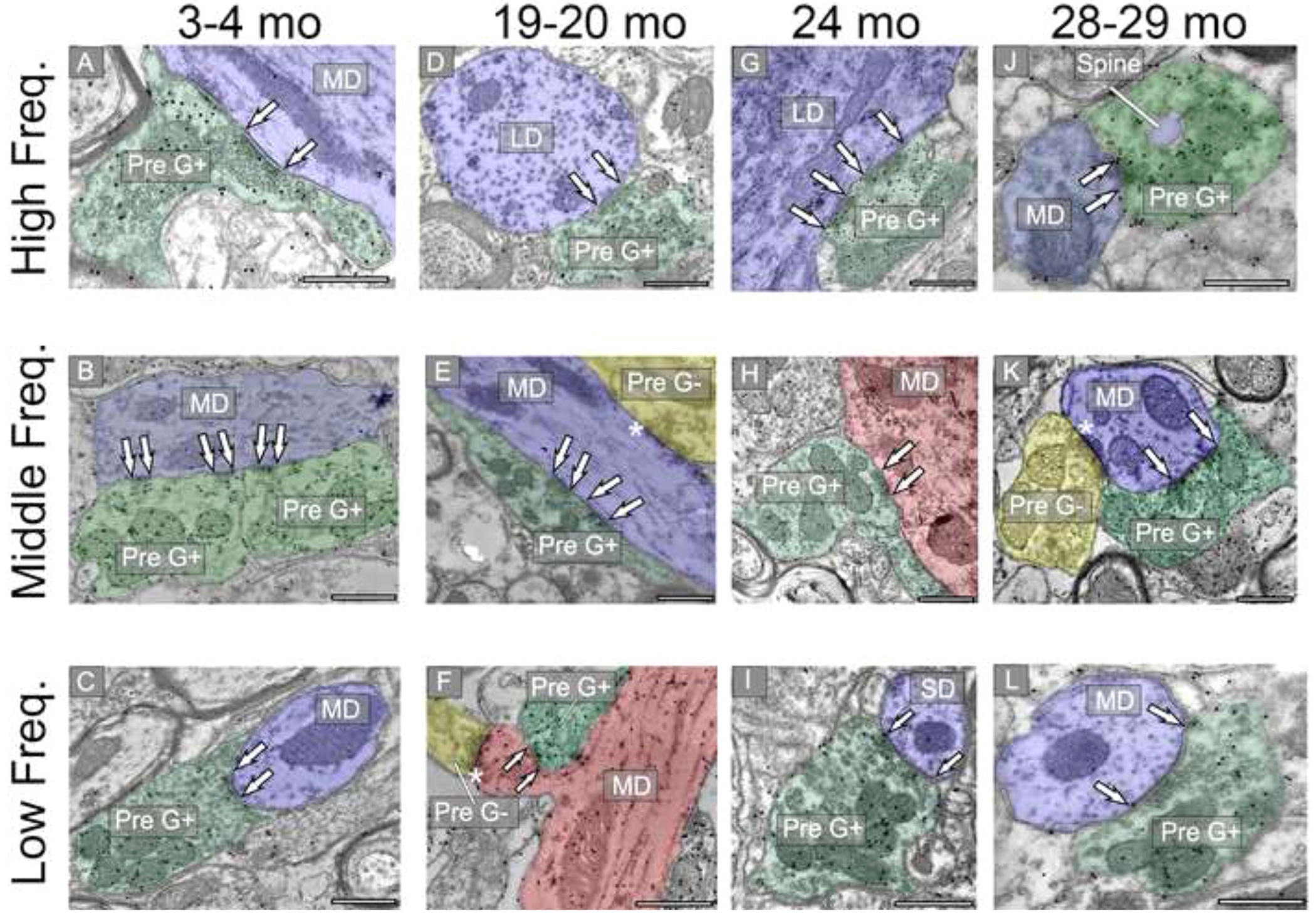
Electron micrographs across four age groups (each age group had five cases) showing GABAergic synaptic ultrastructure across the ventromedial-dorsolateral axis of the central inferior colliculus (ICc). GABAergic presynaptic (Pre G+) terminals are pseudocolored Green. Gabaergic postsynaptic (Post G+) targets are pseudocolored red. Gaba-negative presynaptic (Pre G−) terminals are pseudocolored yellow. Gaba-negative postsynaptic (Post G−) targets are pseudocolored blue. Black dots demonstrate immunogold labeling of GABA. Gabaergic synapses are indicated by pairs of White arrows. Gaba-negative synapses are indicated by an asterisk. **a-c)** electron micrographs showing examples of GABAergic synapses on dendrites across the ventromedial-dorsolateral axis of the ICc in 3–4 month old tissue. **d-f)** electron micrographs showing examples of GABAergic synapses across the ventromedial-dorsolateral axis of the ICc in 19–23 month old tissue. **g-i)** electron micrographs showing examples of GABAergic synapses on dendrites across the ventromedial-dorsolateral axis of the ICc in 24 month old tissue. **j-l)** electron micrographs showing examples of GABAergic synapses on dendrites across the ventromedial-dorsolateral axis of the ICc in 28–29 month old tissue. Ld – large dendrite; MD – medium dendrite; SD – small dendrite. Scale bars = 500 nm.

**Fig. 3.**
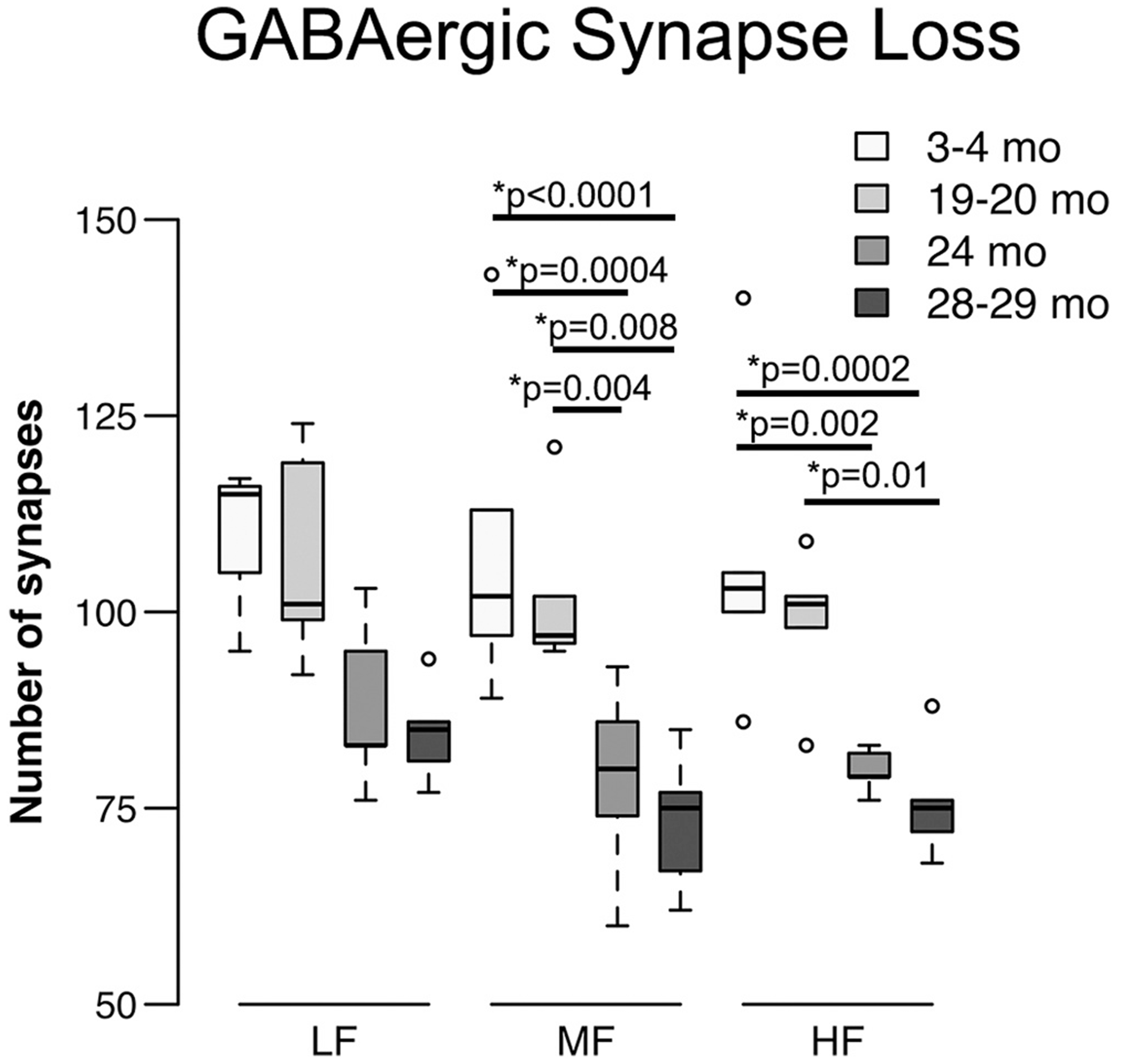
Box plots summarizing the number of GABAergic synapses in the high, middle and low frequency regions of the ICc across four age groups. Broadly, GABAergic synapses are reduced across the IC throughout aging reaching significance at high and middle frequency while nearly reaching significance at low frequency. The most robust and significant decline of GABAergic synapses occurred at high and middle frequency. Pairwise differences demonstrated a significant decrease of GABAergic synapses in the high frequency region between 3 and 4 months and 28–29 months (*p = 0.0002); 3–4 months and 24 months (*p = 0.002); and between 19 and 20 months and 28–29 months (*p = 0.01). pairwise differences also revealed a significant decrease of GABAergc synapses in the middle frequency region between 3 and 4 months and 28–29 months (*p < 0.0001); 3–4 months and 24 months (*p = 0.004); 19–20 months and 28–29 months (*0.008); and 19–20 months and 24 months (*p = 0.004). pairwise differences demonstrated no significant age-related decrease in the number of GABAergic synapses from 3 to 4 months in the high frequency region (19–20 months, p = 0.88); middle frequency region (19–20 months, [p = 0.93]); and low frequency region (19–20 months, [p = 0.99]; 24 months, [p = 0.99]; 28–29 months, [p = 0.06]). in each box plot, dark lines represent the median of the distribution, boxes extend across the interquartile range, and whiskers extend to ± 150 % of the interquartile range. Circles indicate outliers beyond this range. Each age group had five cases.

Presynaptic GABAergic terminals forming synapses were commonly identified across the frequency regions at 3–4 months (Fig. 2A–C, arrow pairs), 19–20 months (Fig. 2D–F, arrow pairs), 24 months (Fig. 2G–I, arrow pairs), and 28–29 months (Fig. 2J–L, arrow pairs). Observing GABAergic and excitatory synapses (Fig. 2E,F,K; asterisks) occurring on the same postsynaptic structure was common. Regardless of location in the ICc and age, GABAergic synapses most commonly target GABA-negative medium sized dendrites (Fig. 2). Significant loss of GABAergic synaptic density was only reached at the middle and high frequencies (Tables 1,2,3; Fig. 3).

#### 3.1.1. GABAergic high frequency loss

At 3–4 months we quantified 534 GABAergic synapses across 16,000 μm^2^ (3.3 synapses/100 μm^2^; Table 1, Fig. 3). At 19–20 months the density of GABAergic synapses was unchanged (3.1 synapses/100 μm^2^; p = 0.88; Table 1, Fig. 3). At 24 months the density of GABAergic synapses reached significance (2.5 synapses/100 μm^2^; p = 0.002; Table 1, Fig. 3). At 28–29 months we quantified 380 GABAergic synapses across 16,000 μm^2^ (2.4 synapses/μm^2^). This was a significant decrease in the density of GABAergic synapses when compared to 3–4 months (*p = 0.0002; Table 1, Fig. 3).

#### 3.1.2. GABAergic middle frequency loss

At 3–4 months we quantified 544 GABAergic synapses across 16,000 μm^2^ (3.4 synapses/100 μm^2^; Table 2, Fig. 3). At 19–20 months the density of GABAergic synapses was not significantly different from 3 to 4 months (3.12 synapses/100 μm^2^; p = 0.94; Table 2, Fig. 3). At 24 months we quantified 393 GABAergic synapses across 16,000 μm^2^ (2.5 synapses/100 μm^2^; Table 2, Fig. 3). This was significantly lower when compared to 3–4 months (*p = 0.0004; Table 2, Fig. 3). At 28–29 months we quantified 366 GABAergic synapses across 16,000 μm^2^ (2.3 synapses/μm^2^). This was a significant decrease in the density of GABAergic synapses when compared to 3–4 months (*p < 0.0001; Table 2, Fig. 3) and 19–20 months (*p = 0.008; Table 2, Fig. 3).

#### 3.1.3. GABAergic low frequency loss

At 3–4 months we quantified 548 GABAergic synapses across 16,000 μm^2^ (3.4 synapses/100 μm^2^; Table 2, Fig. 3). At 19–20 months we quantified 535 GABAergic synapses across 16,000 μm^2^ (3.3 synapses/100 μm^2^) and were not significantly different from 3 to 4 months (p = 0.98; Table 3, Fig. 3). At 24 months we quantified 440 GABAergic synapses across 16,000 μm^2^ (2.8 synapses/100 μm^2^; Table 3, Fig. 3). This was not significantly lower when compared to 3–4 months (p = 1.0; Table 3, Fig. 3) or 19–20 months (p = 1.0; Table 3, Fig. 3. Lastly, 422 GABAergic synapses across 16,000 μm^2^ (2.6 synapses/μm^2^) were quantified at 28–29 months. There was no significant differences when compared to 3–4 months (p = 0.064), 19–20 months (p = 0.153), and 24 months (p = 0.112) (Fig. 3).

### 3.2. GABAergic terminal size

**Fig. 4.**
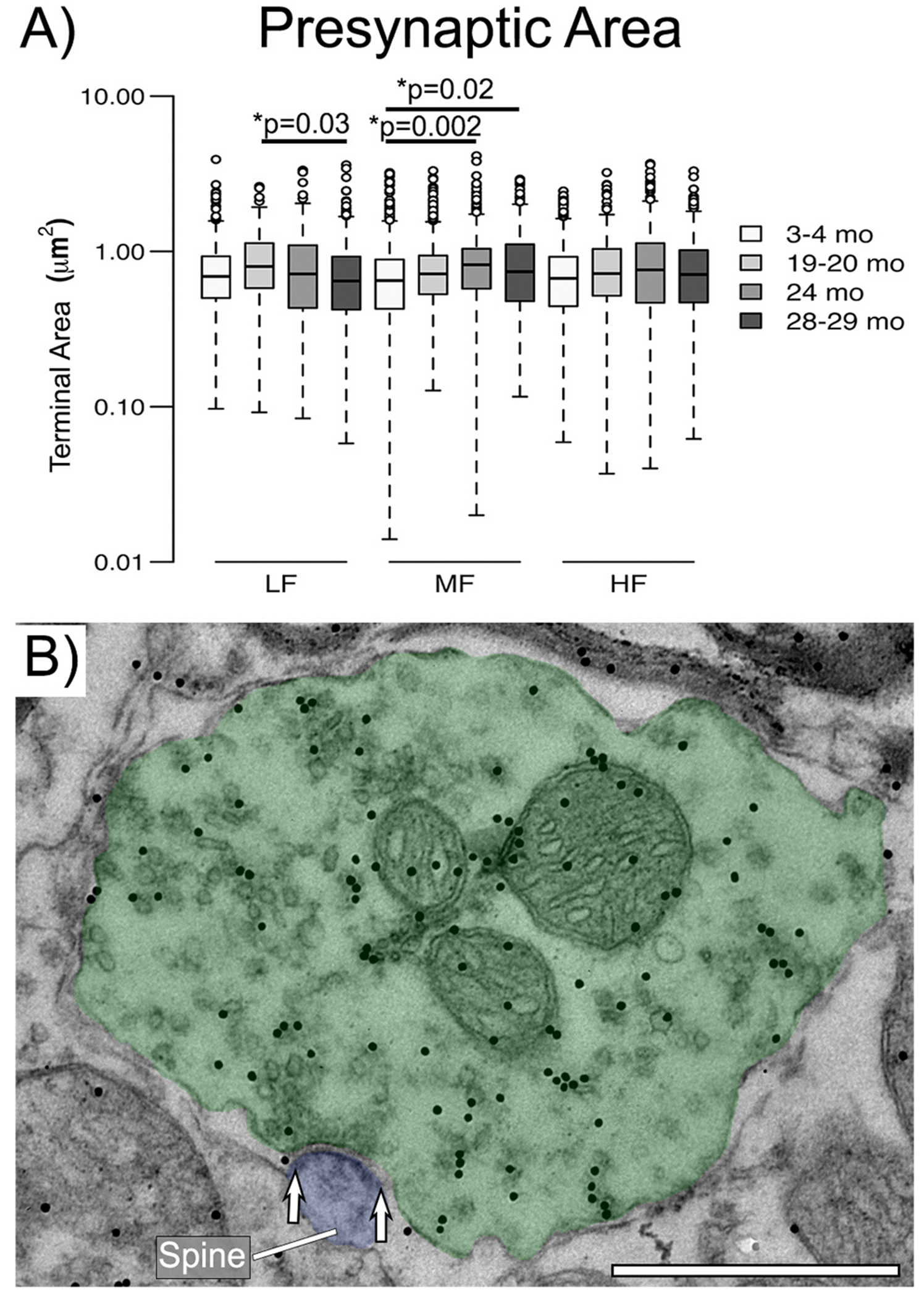
Presynaptic bouton area increases during middle age. **a)** box plots summarizing the average presynaptic bouton area in the high, middle and low frequency regions of the ICc across four age groups. Broadly, presynaptic bouton area increased across the ICc during middle age and then decreased into old age. Pairwise differences demonstrated a significant increase in presynaptic area in the middle frequency region from 3 to 4 months to 24 months (*p = 0.002), and from 3 to 4 months to 28–29 months (*p = 0.02). pairwise differences also revealed a significant decrease in presynaptic bouton area in the low frequency region from 19 to 20 months to 28–29 months (*p = 0.03). pairwise differences demonstrated no significant age-related changes to presynaptic area from 3 to 4 months in the high frequency region (19–20 months, [p = 0.25]; 24 months, [p = 0.12], 28–29 months, [p = 0.25]); middle frequency region (19–20 months, [p = 0.05]); and low frequency region (19–20 months, [p = 0.06]; 24 months, [p = 0.64]; 28–29 months, [p = 0.64]). in each box plot, dark lines represent the median of the distribution, boxes extend across the interquartile range, and whiskers extend to ± 150 % of the interquartile range. Circles indicate outliers beyond this range. Each age group had five cases. Note that the y-axis is plotted on a log scale. **b)** electron micrograph of a large (>1.0 μm^2^) GABAergic presynaptic bouton from the middle frequency region of the central IC at 24 months. The GABAergic presynaptic terminal is pseudocolored Green. A GABA-negative postsynaptic (Post G−) target is pseudocolored blue. Black dots demonstrate immunogold labeling of GABA. A GABAergic synapse is indicated by a pair of White arrows. Black dots demonstrate immunogold labeling of GABA. Scale bar = 500 nm.

We examined the average size of each GABAergic terminal at each age in each region. Terminals with areas of 1.0 μm^2^ or greater were defined as large; 0.5 μm^2^ to 0.99 μm^2^ were medium, and less than 0.5 μm^2^ were small. At 3–4 months the average presynaptic area of GABAergic terminals across the high, middle, and low frequency regions was nearly identical (0.75 μm^2^, HF; 0.74 μm^2^, MF; 0.76 μm^2^, LF (Fig. 4A; Tables 1,2&3). The average size of GABAergic terminals increased in each frequency region with the peak average occurring during 19 months for high and middle and at 24 months for low frequency (Fig. 4A; Tables 1,2&3). It was conspicuous that the larger (>1.0 μm^2^) GABAergic terminals were not downregulated (Fig. 4B). Both small (<0.6 μm^2^) and medium sized terminals were downregulated between each age group in each frequency region. The largest (26 %) loss of small terminals occurred between 3 and 4 months and 19–20 months, while the largest (35 %) loss of medium terminals occurred between 19 and 20 months and 24 months. Terminal area and the diameter of postsynaptic dendrite were positively correlated across most age groups (Table 4). Our linear mixed-effects model found that this increase in GABAergic terminal area was significant in the middle frequency at 24 months (0.9 μm^2^; *p = 0.002) and 28–29 months (0.87 μm^2^; *p = 0.02). Interestingly, a significant decrease in GABAergic terminal size was detected in the low frequency region between 19 and 20 months and 28–29 months (*p = 0.03). Our data suggests that GABAergic downregulation across the aging ICc occurs in a non-uniform fashion and may be driven by sources providing GABAergic terminals that are smaller in area.

### 3.3. Vesicle pools and active zone lengths

**Fig. 5.**
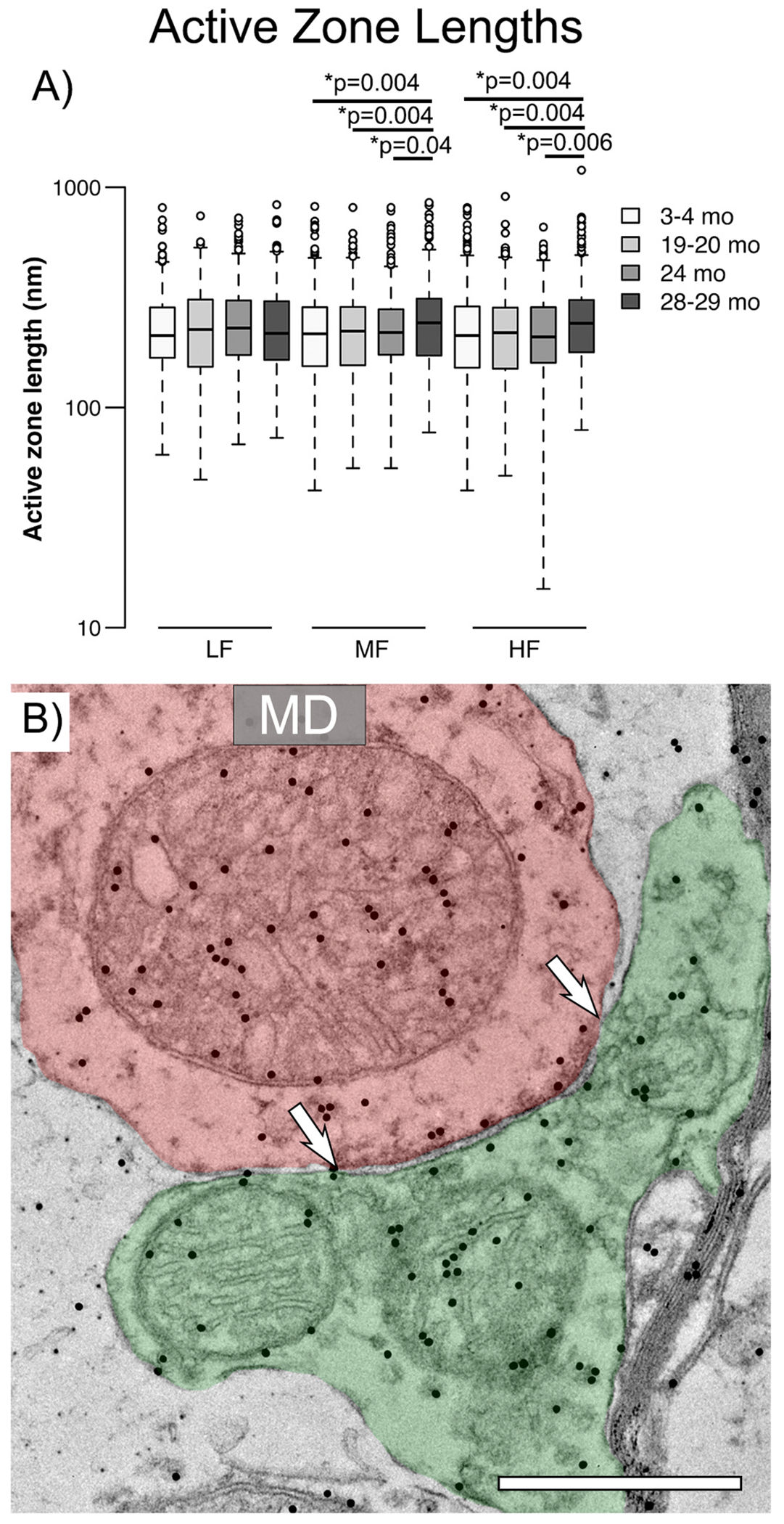
Synaptic length increases with age in the ICc. **a)** box plots summarizing the average synaptic length in the high, middle and low frequency regions of the ICc across four age groups. Pairwise differences demonstrated a significant increase in synaptic length in the high frequency region from 3 to 4 months to 28–29 months (*p = 0.004), 19–20 months to 28–29 months (*p = 0.004), and from 24 months to 28–29 months (*p = 0.006). pairwise differences demonstrated a significant increase in synaptic length in the middle frequency region from 3 to 4 months to 28–29 months (*p = 0.004), 19–20 months to 28–29 months (*p = 0.004), and from 24 months to 28–29 months (*p = 0.04). pairwise differences demonstrated no significant age-related changes from 3 to 4 months in the low frequency region (19–20 months, [p = 0.83]; 24 months, [p = 0.18]; 28–29 months, [p = 0.99]). in each box plot, dark lines represent the median of the distribution, boxes extend across the interquartile range, and whiskers extend to ± 150 % of the interquartile range. Circles indicate outliers beyond this range. Each age group had five cases. Note that the y-axis is plotted on a log scale. **b)** electron micrograph of a 28 month GABAergic presynaptic bouton from the high frequency region of the central IC forming a synapse over 500 nm in length. The GABAergic presynaptic terminal is pseudocolored Green. A GABA-positive postsynaptic target is pseudocolored red. Black dots demonstrate immunogold labeling of GABA. A GABAergic synapse is indicated by a pair of White arrows. Md – medium dendrite. Scale bar = 500 nm.

We quantified the length of each active zone, the number of vesicles that were clustered at the active zone, and the total number of vesicles in each presynaptic synapse. Average active zone lengths were similar across the ICc at 3–4 months (~230 nm, Tables 1,2&3). In general, active zone lengths increased with age across the frequency regions (Fig. 5A). In the high frequency region, the average active zone length at 28–29 months was significantly longer than at 3–4 months (*p = <0.004), 19–20 months (*p = <0.004), and 24 months (*p = 0.006; Fig. 5A). In the middle frequency region, the average active zone length at 28–29 months was significantly longer than at 3–4 months (*p = <0.004), 19–20 months (*p = <0.004), and 24 months (*p = 0.04; Fig. 5A). Active zone lengths in the low frequency did not significantly increase between age groups (Fig. 5A). Fig. 5B is an electron micrograph taken from a 28 month old animal showing a GABAergic presynaptic terminal forming an active zone with a length over 500 nm onto a medium sized GABAergic dendrite.

**Fig. 6.**
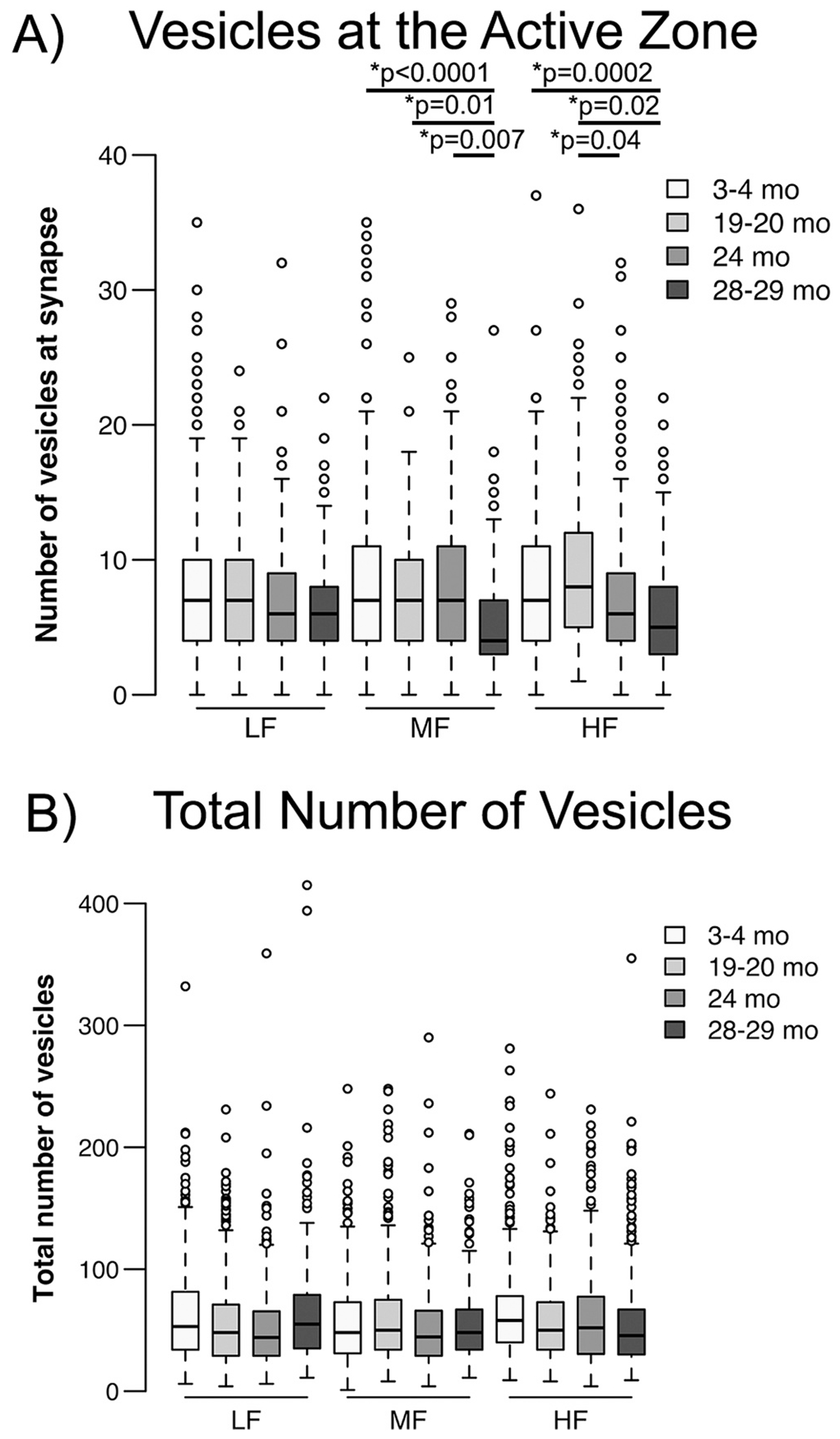
There were less vesicles at the synapses with age in the ICc. **a)** box plots summarizing the average number of vesicles at the synapse in the high, middle and low frequency regions of the ICc across four age groups. Pairwise differences demonstrated a significant decrease in vesicles at the synapse in the high frequency region from 3 to 4 months to 28–29 months (*p = 0.02), 19–20 months to 24 months (*p = 0.04), and from 19 to 20 months to 28–29 months (*p = 0.0002). pairwise differences demonstrated a significant decrease in the middle frequency region from 3 to 4 months to 28–29 months (*p = 0.0001), 19–20 months to 28–29 months (*p = 0.01), and from 24 months to 28–29 months (*p = 0.007). pairwise differences demonstrated no significant age-related changes from 3 to 4 months in the low frequency region (19–20 months, [p = 0.99]; 24 months, [p = 0.73]; 28–29 months, [p = 0.5]). each age group had five cases. **b)** box plots summarizing the average total number of vesicles in the presynaptic bouton in the high, middle and low frequency regions of the ICc across four age groups. Pairwise differences demonstrated no significant age-related changes across any age in any region. In each box plot, dark lines represent the median of the distribution, boxes extend across the interquartile range, and whiskers extend to ± 150 % of the interquartile range. Circles indicate outliers beyond this range. Each age group had five cases.

We found that the number of vesicles at the active zone decreases with age in the ICc. We found significant declines in the high and middle frequency regions (Fig. 6A). In the high frequency region, the average number of vesicles at the active zone was 8.1 (Table 1). The average dropped to 5.9 at 28–29 months (*p = 0.02; Fig. 6a, Table 1). The average number of vesicles at the active zone for 19–20 months increased to 9.5 (Fig. 6A; Table 1). This was significantly higher than 24 months (*p = 0.04) and 28–29 months (*p = 0.02; Fig. 6A, Table 1). In the middle frequency region, the average number of vesicles at the active zone for 3–4 months, 19–20 months, and 24 months was 8.7, 7.3, and 7.7, respectively (Fig. 6A; Table 2). Each of these values were significantly higher than the 5.1 average at 28–29 months (Fig. 6A; Table 2). In the low frequency region, the number of vesicles at the active zone trended downward, however no significance was detected. (Fig. 6A).

Lastly, in each ICc region the total number of vesicles in each presynaptic terminal was quantified. Broadly, the main finding was that the average vesicle pool size did not change with age in any region (Fig. 6B). This non-significance may be driven by variability within each age group and each region (compare Tables 1,2&3). Regardless of age, the total vesicle pool had positive correlation with the diameter of the postsynaptic target (Table 4). Most cases regardless of age and region had terminals that contained between 50 and 70 vesicles. Ultimately it appears that aging may not affect the overall vesicle pool in each GABAergic terminal, however the number of vesicles at the active zone is reduced.

### 3.4. Mitochondria ultrastructure

In our previous report on synaptic changes in the lateral cortex of the IC (IClc) we observed that mitochondria have increasingly poor ultrastructure with age (Mafi et al., 2022). Thus, we wanted to determine if aging had the same effect in the ICc. As we had previously seen with GABAergic terminals in the IClc, there was no difference in the average number of mitochondria with age (Tables 1,2&3). Perhaps unsurprisingly, correlation coefficients demonstrated that the size of the terminal area was positively correlated with the presence of presynaptic mitochondria at each age group (Table 4). In the high frequency region, there was an average of 1.4 mitochondria at 3–4 months and 1.5 mitochondria at 28–29 months (Table 1). In the middle frequency region, there was an average of 1.6 mitochondria at 3–4 months and 1.5 mitochondria at 28–29 months (Table 2). In the low frequency region, there was an average of 1.5 mitochondria at 3–4 months and 1.4 mitochondria at 28–29 months (Table 3).

**Fig. 7.**
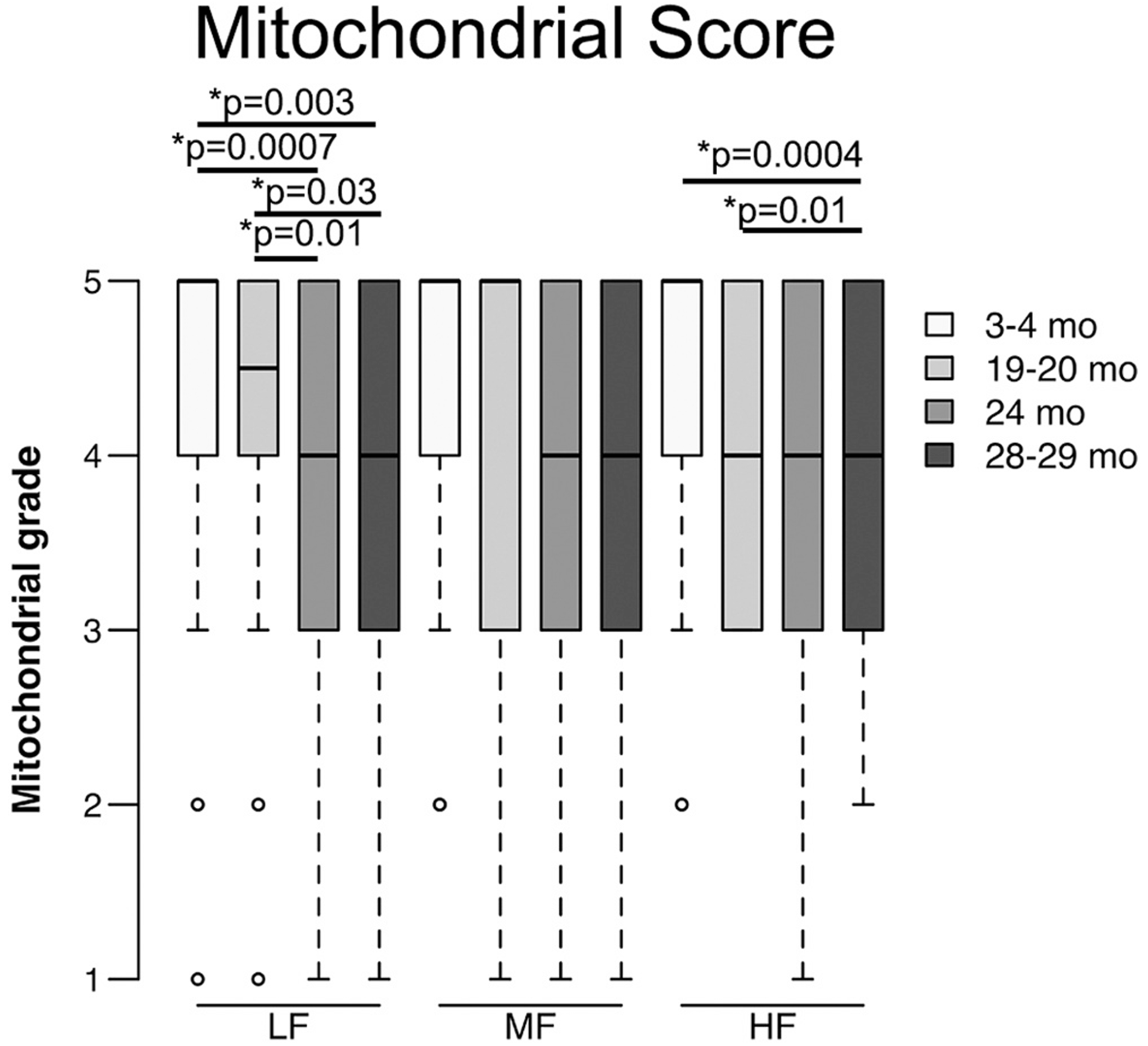
Mitochondria ultrastructural scores decline with age in the ICc. box plots summarizing the mitochondrial scores in the high, middle and low frequency regions of the ICc across four age groups. The most robust and significant decline in mitochondrial score occurred at high and low frequency. Pairwise differences demonstrated a significant decrease of scores in the high frequency region between 3 and 4 months and 28–29 months (*p = 0.0004) and between 19 and 20 months and 28–29 months (*p = 0.01). pairwise differences demonstrated a significant decrease of scores in the low frequency region between 3 and 4 months and 24 months (*p = 0.0007), 3–4 months and 28–29 months (*p = 0.003), 19–20 months and 24 months (*p = 0.01), and 19–20 months and 28–29 months (*p = 0.03). pairwise differences did not reveal a significant difference in mitochondria scores from 3 to 4 months in the middle frequency region (19–20 months, [p = 0.98]; 24 months, [p = 0.14]); 28–29 months, [p = 0.11]). In each box plot, dark lines represent the median of the distribution, boxes extend across the interquartile range, and whiskers extend to ± 150 % of the interquartile range. Circles indicate outliers beyond this range. Each age group had five cases.

The ultrastructure of each presynaptic mitochondria was scored (5 being excellent; 1 being poor) reflecting the intactness of its membranes and cristae. The general trend was that the mitochondria scoring declined with age (Fig. 7). Although the number of presynaptic mitochondria and terminal area was positively correlated, the mitochondrial grades were negatively correlated with terminal size in early life and became more positively correlated later the later age groups (Table 4). Mitochondria grade was also negatively correlated with active zone length at each age (Table 4). Interestingly, significance was reached in the high and low frequency regions, but not the middle frequency (Fig. 7). In the high frequency region, the average mitochondria score was 3.6 at 28–29 months, which was significantly lower than 3–4 months (4.4; *p = 0.0004) and 19–20 months (4.2; *p = 0.01). In the low frequency region, the average mitochondria score was 3.6 at 24 months, which was significantly lower than 3–4 months (4.4; *p = 0.0007) and 19–20 months (4.2; *p = 0.01). The average mitochondria score in the low frequency region at 28–29 month was 3.7 which was significantly lower than 3–4 months (4.4; *p = 0.003) and 19–20 months (4.2; *p = 0.03). It is interesting to note that the low frequency was the only region with significant mitochondrial ultrastructure decline at 24 months, which is when this strain of rat is known to have elevated thresholds for lower frequencies.

### 3.5. Postsynaptic targeting

**Fig. 8.**
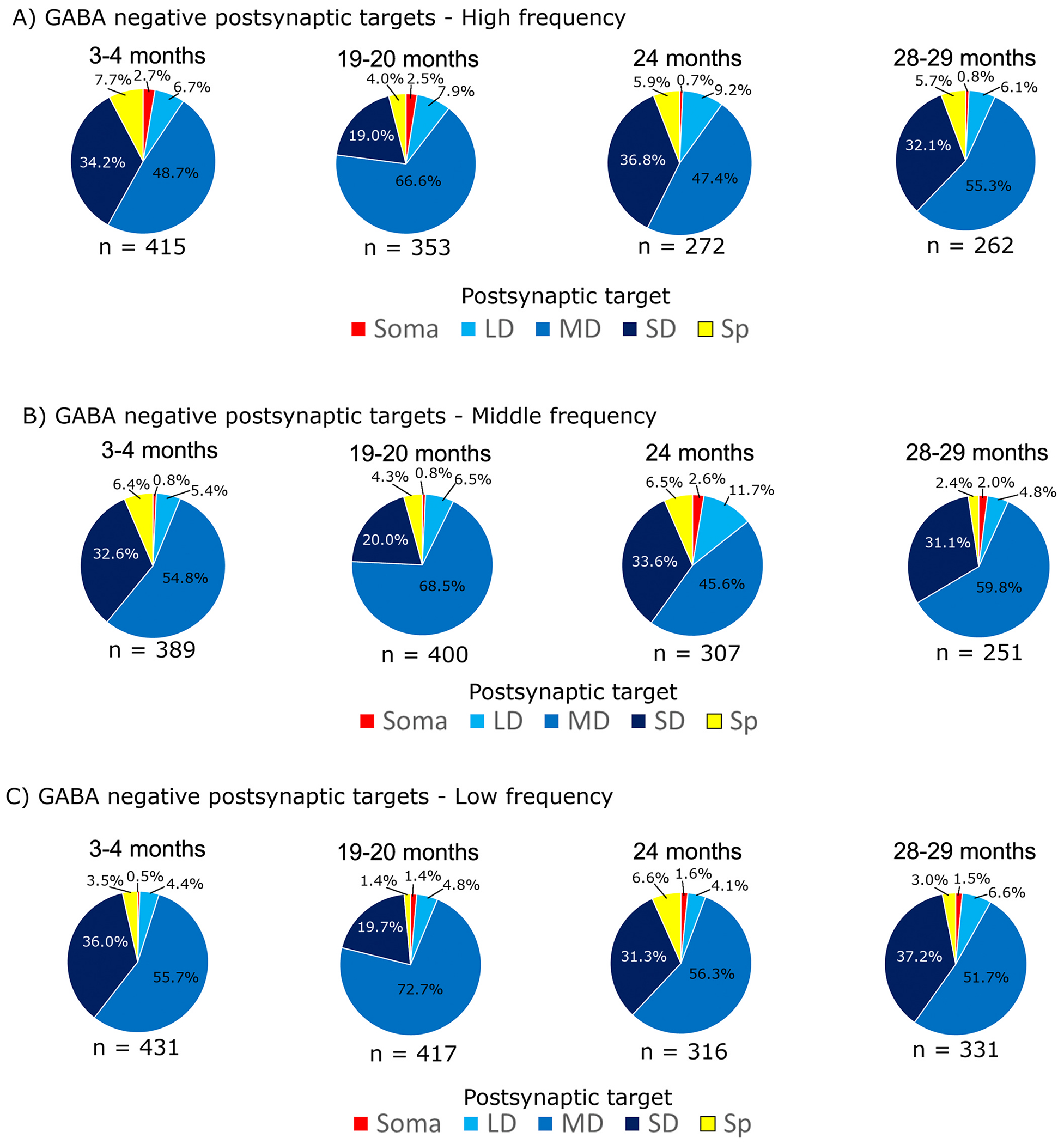
Pie charts showing the distribution of GABAergic terminals forming synapses onto GABA-negative postsynaptic targets across four age groups of the ICc. A) pie charts showing the frequency with which GABAergic terminals formed synapses onto GABA-negative postsynaptic structures in the high frequency region. B) pie charts showing the frequency with which GABAergic terminals formed synapses onto GABA-negative postsynaptic structures in the middle frequency region. C) pie charts showing the frequency with which GABAergic terminals formed synapses onto GABA-negative postsynaptic structures in the low frequency region. Regardless of frequency region, the proportion of synapses on medium sized dendrites increases at 19–20 months, and then decreases at 24 months. Each age group had five cases.

**Fig. 9.**
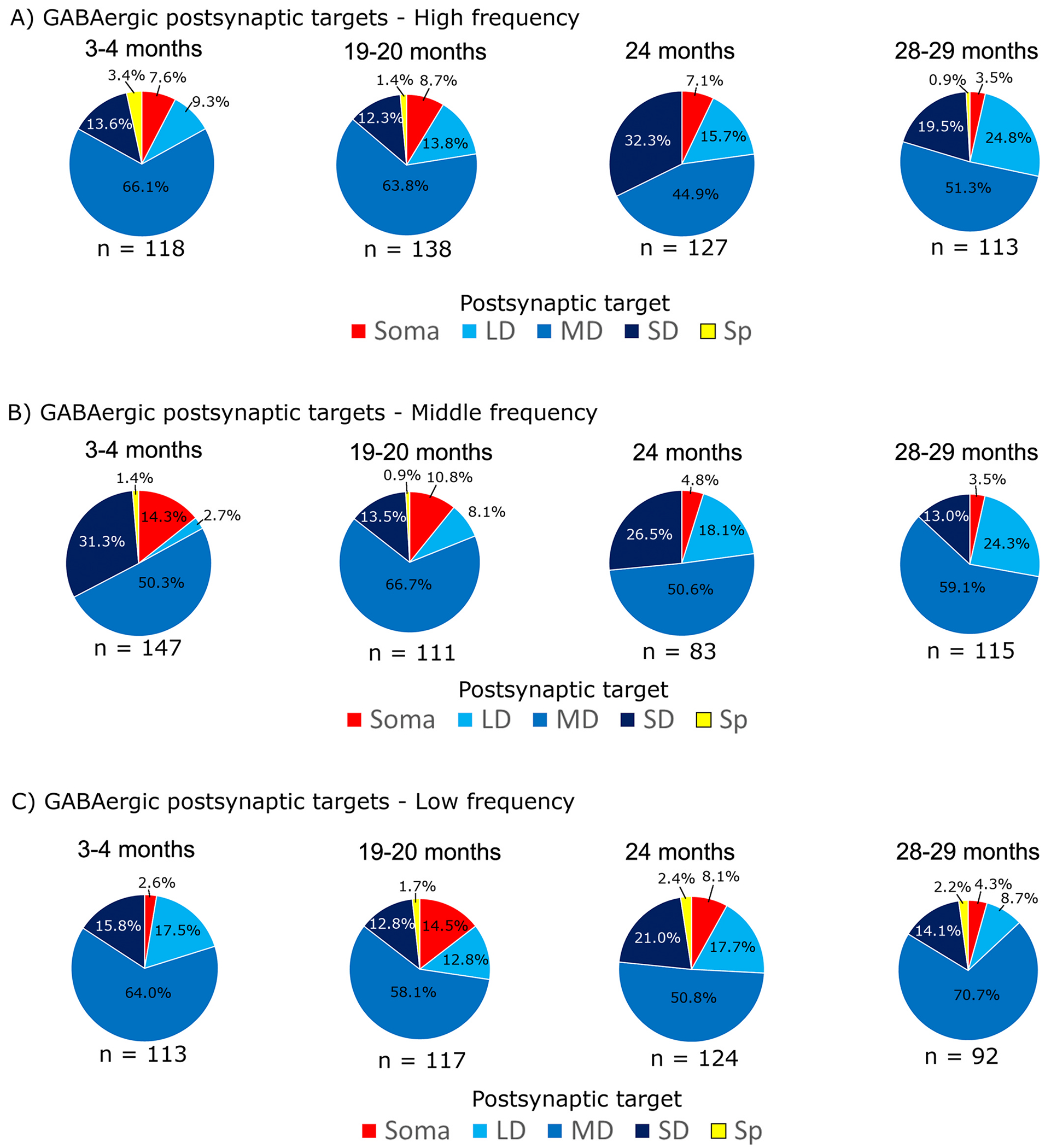
Pie charts showing the distribution of GABAergic terminals forming synapses onto GABAergic postsynaptic targets across four age groups of the ICc. A) pie charts showing the frequency with which GABAergic terminals formed synapses onto GABAergic postsynaptic structures in the high frequency region. B) pie charts showing the frequency with which GABAergic terminals formed synapses onto GABAergic postsynaptic structures in the middle frequency region. C) pie charts showing the frequency with which GABAergic terminals formed synapses onto GABAergic postsynaptic structures in the low frequency region. Each age group had five cases.

We recorded the postsynaptic target for each GABAergic synapse (Fig. 8&9). Postsynaptic targets included GABA-negative and GABAergic somas, dendrites, spines and boutons. Most synapses occurred on a medium or small dendrite (Fig. 8&9). As previously mentioned, dendrites were categorized by their diameters as large (>1.5 μm), medium (0.5–1.5 μm) and small (<0.5 μm). Of the 5565 GABAergic synapses, 23 targeted a bouton. This represents less than 1 % of the quantified synapses, thus we did not find value representing them in Fig. 8&9. Most (15/23) of these postsynaptic boutons were classified as GABAergic.

#### 3.5.1. GABA-negative postsynaptic targets

In total, 4144 (75 %) of the GABAergic terminals formed synapses with a GABA-negative dendrite, spine, and soma (Fig. 8). It was conspicuous that regardless of age the synaptic distribution across the frequency regions was not qualitatively different (Fig. 8). The distribution of these synapses at 3–4 months occurred mostly (48–56 %) on medium dendrites (Fig. 8). At 19–20 months there was noticeable increase of synapses further targeting medium sized dendrites with each frequency region at 66–73 % (Fig. 8). This increase underscored that the age-related loss of synapses occurred largely on small dendrites (Fig. 8). Interestingly, at 24 months and 28 months the distribution of the remaining synapses was similar to 3–4 months (Fig. 8). Thus, perhaps there is a two-stage loss of GABAergic synapses that target GABA-negative dendrites such that the initial loss at 19 months is on smaller caliber dendrites followed by loss at 24 months on medium caliber dendrites.

#### 3.5.2. GABAergic postsynaptic targets

**Fig. 10.**
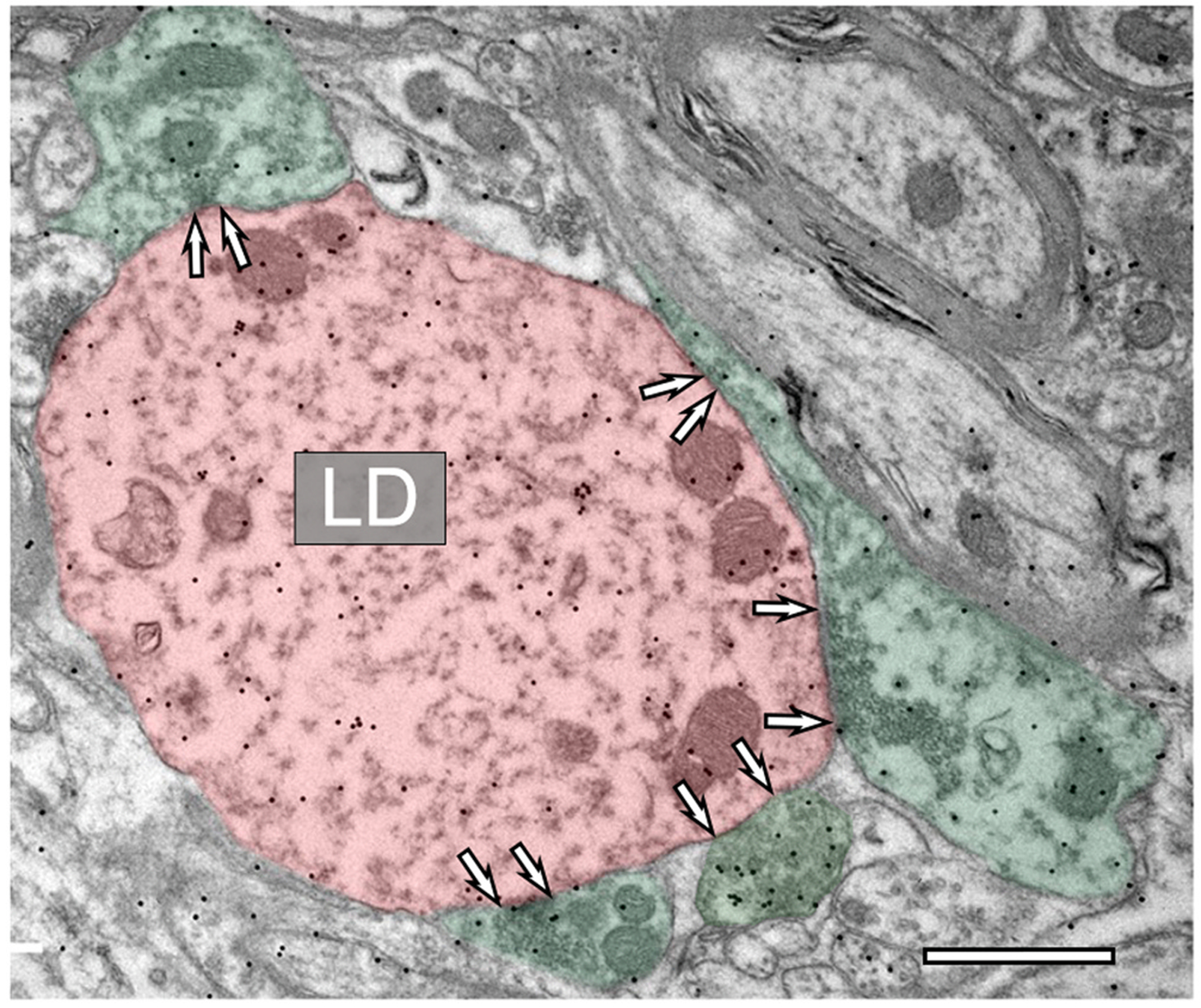
Electron micrograph showing a 28–29 month old GABAergic dendrite (red) from the high frequency region that is receiving input from four GABAergic terminals (Green) that form a total of five symmetric synapses (arrow pairs). black dots demonstrate immunogold labeling of GABA. Ld – large dendrite. Scale bar = 500 nm.

In total, 1398 (25 %) of the GABAergic terminals formed synapses with a GABAergic dendrite, spine, and soma (Fig. 9). The age-related loss of synapses in the high frequency region was only 4 %, while the loss was 22 % and 19 % across the middle and low frequency regions, respectively (Fig. 9). These synapses also preferentially targeted medium caliber dendrites regardless of location and age (compare Fig. 8&9). A few baseline observations stood out: 1) postsynaptic GABAergic somas and large dendrites were proportionally more common than postsynaptic GABA-negative somas and large dendrites, and 2) postsynaptic GABAergic spines were uncommon and, in some age-region combinations, not observed (Fig. 9). In each frequency region changes in distribution were robust at 24 months in that fewer medium dendrites were targeted (Fig. 9). Perhaps the most interesting finding was how GABAergic synapses were or were not redistributed onto large caliber GABAergic dendrites with age. In the high and middle frequency regions the proportion of synapses on large dendrites increased throughout aging and at 28–29 months was nearly 25 % (Fig. 9&10). Large GABAergic dendrites at 28–29-month-old were more commonly observed with multiple GABAergic inputs (Fig. 10). It is interesting to note that postsynaptic mitochondria were often observed around the perimeter of the dendrite, near the active zones of these GABAergic synapses (Fig. 10). However, in the low frequency region, the number of synapses targeting large caliber GABAergic dendrites decreased (17.7–8.7 %) from 24 months to 28–29 months (Fig. 9).

## 4. Discussion

The current study examines the age-related changes to GABAergic synaptic ultrastructure in the high, middle, and low frequency regions of the ICc. Our primary finding was that the density of GABAergic synapses was lost with age, however the loss was not uniform across the ICc. The greatest loss of GABAergic synapses occurred in the middle and high frequency regions at 28–29 months. There was a loss of GABAergic synapses in the low frequency region, however it was not as robust nor was significance reached by our analysis. Examination of the remaining GABAergic synapses yielded several findings. First, presynaptic bouton areas were larger on average during middle age, with a significant reduction in area between middle and old age in the low frequency region. Second, the active zones in the high and middle frequency regions were significantly longer at 28–29 months. Third, the average number of vesicles at the synapse was lower in the high and middle frequency regions. Fourth, mitochondrial ultrastructure was degraded across the ICc with the most significant changes occurring across middle and old age in the low frequency region. Fifth, we ran correlations between the ultrastructural criteria that we quantified and found that most correlation patterns differed across age groups and thus these relationships are not likely uniform throughout life. Lastly, and perhaps most importantly, most of the lost GABAergic synapses targeted GABA-negative dendrites. This may allow hyperexcitability, via disinhibition, to increase throughout the numerous IC circuits. Our data also suggests that synaptic rearrangement occurs throughout aging with GABAergic synapses targeting fewer GABA-negative small caliber dendrites at 19–20 months, and then targeting fewer medium size dendrites at 24 months. GABAergic synapses targeting GABAergic dendrites also demonstrated differences with age. Large GABAergic dendrites received more GABAergic inputs at old age in the high and middle frequency regions. Overall, several ultrastructural changes occurred in the low frequency region that were not as robust or consistent across the rest of the IC. In the next section we address technical considerations that were made to analyze our data set.

### 4.1. Technical considerations

In the current and our previous studies, we use the FBN rat as it has a longer median lifespan than other strains of mice and rats and is a recommended aging model by National Institute on Aging (Lipman et al., 1996; Lipman, 1997). Furthermore, the FBN/Fischer-344 rat models are often used to characterize the aging central auditory system, often with a focus on GABAergic neurotransmission (Caspary et al., 2008; Burianova et al., 2009; Syka, 2010; Parthasarathy and Bartlett, 2011; Caspary and Llano, 2018; Cai et al., 2018; Robinson et al., 2019; Mafi et al., 2020, 2021, 2022; Syka, 2020; Kommajosyula et al., 2021; Koehler et al., 2023). Our 3–4 month age group is a standard “young” group when hearing is intact and no known deficits to hearing abilities are known to occur. The 28–29 month age group has been characterized to have significant hearing loss (Cai et al., 2018). The two middle age groups, 19–20 months and 24 months, reflect ages when hearing deficits are not commonly reported in the FBN rat and when hearing thresholds begin to be significantly elevated, respectively (Cai et al., 2018). However, in the current study the rats’ hearing thresholds were not measured, and we do not fundamentally know if one of our 24-month-old rats had poorer or better hearing than a given 19 month old rat. Thus, the lack of a functional assay is an obvious weakness of the current study. We equate anatomical regions across the dorsolateral to ventromedial axis of the ICc with tonotopic axis of the ICc to address frequency regions (e.g. the ventromedial-most block is equated with the high-frequency region). We did not characterize IC cells with electrophysiological techniques in the current study. We took the ventromedial-dorsomedial axis of the ICc tissue and divided it into thirds. While we do not know the characteristic frequencies represented in each tissue block, as the tonotopic axis of the IC is well established, we have no reason to believe that our ventromedial most block does not represent higher frequencies compared to the other two blocks; and the dorsolateral most block would represent the lowest frequencies. An important consideration in the interpretation of our data is that, although a common view of the IC’s tonotopic map is one of a continuous gradient, the rat IC tonotopic map is organized in a discontinuous stepwise manner (Malmierca et al., 2008). Lastly, as mentioned in the Methods, the exact border, if there is one, between ICc and ICd is not well defined. As such, the tissue from the dorsolateral IC is trimmed out with a 0.2 mm buffer with the intent to reduce the risk of including ICd. Taken together, our ICc blocks may represent frequencies closer to one end of the tonotopic map than the other. Regardless, our data demonstrates non-uniform changes across the dorsolateral-ventromedial axis of the ICc.

We employ immunogold-EM to detect GABAergic profiles. While it is a very sensitive technique and the gold standard of determining the neurochemical profile of ultrastructure with TEM, small GABAergic profiles may be difficult to conclusively label. First, GABA has greater expression in the terminals and axons than smaller caliber dendrites. Second, spine quantification with TEM may not be straightforward as 1) the parent dendrite may not be visible, 2) spine apparatus may not be visible, and 3) the lack of microtubules may not be obvious in smaller profiles when sectioned at oblique angles (Rockel and Jones, 1973; Ribak and Roberts, 1986; Paloff and Usunoff, 1992; Nakamoto et. al, 2014). While TEM has many strengths (e.g. greatest resolution), a weakness is that that our data is derived from cross sectional images rather than from volumetric reconstructions. Thus, our data is not reflecting the entirety of a presynaptic terminal and does not capture the area of a given synapse.

### 4.2. Comparisons to previous studies

One of the motivations to conduct the current study was to expand upon the seminal work of Helfert et al. (1999). In Helfert et al., (1999) they examined the aging ICc from the “mid-frequency” area. Given that the nature of hearing loss commonly favors one end of the auditory spectra in aged mammals, we wanted to expand our knowledge of the age-related ultrastructural changes across a wider representation of 1) the ICc axis and, 2) age groups. Our analysis appears to be in line with what was previously reported in that the loss of GABAergic synapses at old age in the middle frequency region in both the current study and Helfert et al., (1999) was ~33 %. Regardless of aging, presynaptic GABAergic ultrastructure across the ICc axis in the current study had characteristics that agreed with previous reports in different species and IC subdivisions in that: 1) terminals that formed a symmetric PSD routinely had flattened/pleomorphic shaped vesicles, 2) most (~85 %) GABAergic terminals were < 0.99 μm^2^ (which previous reports characterize as small), 3) most (>90 %) GABAergic terminals made synapses with dendrites (as opposed to spines and somas), and 4) most (~70 %) GABAergic terminals targeted non-GABAergic neurons (Rockel and Jones, 1973; Oliver and Morest, 1984; Ribak and Robertss, 1986; Robertss and Ribak, 1987; Paloff and Usunoff, 1992; Helfert et al., 1999; Nakamoto et al., 2013; Nakamoto et al., 2014; Mafi et al., 2022; Mellott et al., 2024). It appears that many ultrastructural characteristics of presynaptic GABAergic terminals are preserved 1) across species, 2) tonotopic axis of the ICc, and 3) IC subdivisions.

We recently reported on the aging ultrastructure of layer III of the IClc (Mafi et al., 2022). For reference, the tissue blocks in Mafi et al., (2022) were taken at the dorsoventral midpoint within layer III, which represents the midfrequency region of lemniscal input to the IClc (Oliver, 2005; Loftus et. al., 2008). At a young age, the density of GABAergic synapses was ~10–15 % greater in the ICc than the IClc. Loss of GABAergic synapses was very similar between our previous study and the current study in that significance was reached at 28–29 months and the reduction in IClc was ~28–30 % (Mafi et al., 2022). Several other results were similar across the two studies in that 1) GABAergic terminal areas increased with age, mitochondria ultrastructure worsened with age, and GABAergic terminals predominately targeted medium and small dendrites. However, proportionally, GABAergic terminals more commonly targeted large GABAergic dendrites in the IClc than in the ICc (Mafi et al., 2022). It is interesting to note that in the current study that the correlation between terminal area and mitochondria grade progressed from a significant negative correlation at our youngest age group to more positive correlations during aging. We interpret this to mean that as synapses are lost with aging, the surviving terminals are more likely to have mitochondria with greater structural intactness. Thus, the functional integrity of mitochondria may play a role in the preservation of select presynaptic terminals as these circuits age. One of the starker differences between the study is that the GABAergic active zones in the IClc did not lengthen as they did at 28–29 months in the current study. Excitatory active zone lengthening has been noted as a compensatory mechanism (Levine et al., 1988; Helfert et al., 1999). We are unaware of reports of GABAergic active zone lengthening, but perhaps this is also a compensatory attempt related to additional neurotransmitter release. Another stark difference was that in the IClc we found that mitochondria grade and active zone length were strongly correlated in our oldest group (Mafi et al., 2022). In the current study, the correlation between grouped mitochondria grades and active zone length was consistently weak across the age groups. This difference may reflect differences in the framework of GABAergic input to the lemniscal and non-lemniscal IC. Perhaps, aging differentially affects ICc mitochondria and mitophagy in presynaptic terminals originating from intrinsic, extrinsic (e.g. dorsal nucleus of the lateral lemniscal), and commissural sources.

Aging EM studies in mice have reported that strains (C57BL/6J) with elevated hearing thresholds due to aging lose axosomatic inputs to principal cells in the IC, while strains (CBA) that maintain normal hearing thresholds during aging do not undergo such loss (Kazee et al., 1995; Kazee and West, 1999). Given 1) the manner in that we imaged the IC tissue to keep elements of the study random and, 2) most synapses in the IC neuropil are not on a soma; we did not capture numerous examples of axosomatic inputs onto singular somas. It is interesting that previous studies of axosomatic inputs of the IC vary in their findings. In cat and rat, large IC somas receive dense axosomatic inputs, while medium and smaller somata receive far fewer inputs (Oliver, 1984; Ribak and Roberts, 1986; Paloff et al., 1992; Helfert et al., 1999; Paloff et al., 2004). In mice, axosomatic inputs appear to be far fewer than in rat and cat as the membrane of a given soma is rarely (<5 %) contacted by these inputs (Kazee et al., 1995). EM reports in cat and rat found that axosomatic input to large IC cells form predominately symmetric PSDs with pleomorphic/flattened vesicles (Ribak and Roberts, 1986; Paloff et al., 1992). Thus, large IC somas receive more inhibitory inputs than excitatory ones. Interestingly, a subset of large GABAergic somas was discovered to have a specialized construct of excitatory inputs (Ito et al., 2009; Beebe et al., 2016; 2018). We agree with Kazee et al., (1995) that in a model that develops age-related hearing loss, synaptic loss across the frequency domains of the IC is prominent. However, in the context of just axosomatic contacts it was largely unchanged in the current study. If anything, there was a gain of GABAergic synapses on GABAergic somas in the low frequency, and what was previously reported (Helfert et al., 1999).

There is an interesting synaptic rearrangement that occurs in the aging IC. Like the previous reports on the aging IC ultrastructure, we find that in the high and middle frequency regions that there is a greater occurrence of GABAergic synapses targeting large GABAergic dendrites with age (compare Fig. 8&9; Helfert et al., 1999; Mafi et al., 2022). However, this observation does not hold true in the low frequency region as there is a reduction of GABAergic terminals forming synapses with large GABAergic dendrites. How these data translate functionally to an animal model that loses low frequency hearing first with age is curious. In Helfert et al., (1999) it was postulated that the decrease in synaptic density in the aging IC was in response to a similar age-related pruning in dendritic branches. While quantifying each dendrite was not a goal of the current study, our unpublished observations indicate a greater loss of dendrites in the high frequency region (~20 %) as compared to the low frequency region (~10 %). It is worth noting that intralaminar compartments in the rat IC appear to be absent at low frequencies, which has led to the suggestion that stellate/less flat cells, as opposed to disc-shaped/flat cells, are rarer in the low frequency domains (Malmierca et al., 1993, 1995, 2005). As we found that the most robust age-related loss of GABAergic synapses occurred in the aged high and middle frequency regions, perhaps the increased loss of these synapses was targeting stellate cells. However, it is important to remind the reader that to better ensure our low frequency block was not invading non-lemniscal IC regions, we trimmed the blocks out with a small buffer, thus it is unlikely that our low frequency block represents the lowest possible frequencies processed by the ICc. Future studies will hopefully determine the specific postsynaptic cell types that lose these GABAergic inputs.

The IC receives GABAergic inhibition from intrinsic sources, the commissural IC, each subdivision of the lateral lemniscus, and some smaller populations of GABAergic cells in the superior olivary complex and cochlear nucleus (Oliver, 1985, 1987; Shneiderman et al., 1988; Shneiderman and Oliver, 1989; González-Hernández et al., 1996). We were surprised that most of our data collected (synaptic density, bouton size, active zone length, vesicle numbers) at 2–3 months was indistinguishable between high, middle and low frequency (compare Tables 1, 2&3). We are reporting averages, and thus not accounting for the many GABAergic inputs to the IC, but it appears that the organizational details of GABAergic inputs may be conserved across the ICc. An interesting finding from the current study was that the presynaptic terminal area significantly decreased at the low frequency region from middle to old age. This significance was driven by a reduction in the density of larger (>1.0 μm^2^) GABAergic presynaptic terminals. Given that our animal model develops low frequency presbycusis (Keithley et al., 1992; Cai et al., 2018), perhaps these lost large terminals underlie their degrading hearing abilities. Reports across a few species suggest that large GABAergic terminals may originate from lemniscal sources as the larger GABAergic terminals are predominantly found in the ICc (Oliver and Shneiderman and Oliver, 1989; Oliver et al., 1995; Nakamoto et al., 2014). A potential candidate of this lost source of larger GABAergic terminals is the dorsal nucleus of the lateral lemniscus (DNLL). The DNLL is a GABAergic nucleus that robustly projects to the ipsilateral and contralateral IC in a topographic and laminar organization and is functionally critical to binaural and temporal processing (Kudo, 1981; Shneiderman and Oliver, 1989; Kelly et al., 1998; Bajo et al., 1999). Perhaps it is the loss of these organized inputs from the low frequency region of the DNLL to the low frequency region of the ICc that contributes to the increasing hearing thresholds at low frequency that initially occurs in the FBN rat. It will be imperative to determine the source of these large lost inputs and whether the loss is mirrored in high frequency regions in models that develop high frequency presbycusis.

### 4.3. Functional implications

The current study demonstrates a significant age-related loss of GABAergic synapses in the high and middle frequency regions in an animal model that develops low frequency presbycusis. Across our four age groups the most conspicuous decline in GABAergic synaptic density occurred at 24 months, the age that FBN rats begin to have elevated hearing thresholds. However, given the development of low frequency presbycusis, we hypothesize that a general loss of synapses in the ICc does not equate to the onset of age-related hearing loss. Helfert et al., (1999) found a significant loss of GABAergic synapses in the middle frequency region. They also found a complementary loss of excitatory input and concluded that the balance of inhibition and excitation, and synaptic organization, to a given cell is little changed. At the very least, the age-related loss of synapses in the mid-ICc likely does not affect the response to simple acoustic stimuli (Palombi and Caspary, 1996a,b). Helfert et al., (1999) proposed a model of age-related synaptic changes across a disc-shaped neuron in the ICc. The model illustrates a loss of GABAergic synapses on secondary and tertiary dendrites, while there is an increase of GABAergic inputs to proximal dendrites. The current study agrees with this model; however, the model is specific to disc-shaped cells of the IC as stellate cells were not distinguishable and could not be considered (Helfert et al., 1999). We found GABAergic synaptic loss in the low frequency region, where stellate cells are not as common, was not significant as compared to the rest of the ICc. This may imply that aging differentially affects the functions of ICc cell types. Recently it was discovered that subpopulations of IC stellate cells express 1) neuropeptide Y (NPY) and are inhibitory, or 2) vasoactive intestinal peptide and are excitatory (Goyer et al., 2019; Silveira et al., 2020, 2023). Determining whether aging downregulates or spares NPY releasing GABAergic terminals in the IC will give insight to which specific inhibitory and excitatory circuits are affected before and after the onset of age-related hearing loss.

There is a robust age-related GABAergic downregulation in the aged IC (Caspary et al., 2008; Syka, 2020). In general, the loss of GABA in the IC has been viewed both as a compensatory and maladaptive mechanism. The initial loss of GABA is likely a homeostatic response to the degraded periphery and diminished excitation to the central auditory system (Richardson et al., 2012; Caspary et al., 2008; Caspary and Llano, 2018). However, at some point in life the loss of GABAergic tone in the IC may contribute to increases in central gain, hyperexcitable microenvironments, poor speech perception, a reduction in nonmonotonic rate-intensity functions, and/or poorer temporal processing within complex/noisy environments; ultimately leading to tinnitus, hyperacusis, and presbycusis (Willott et al., 1988a, 1988b; Palombi and Caspary, 1996; Frisina, 2001; Brozoski et al., 2002; Walton et al., 2002; Norena, 2011, Parthasarathy and Bartlett, 2011; Rabang et al., 2012; Auerbach et al., 2014; 2019; Parthasarathy et al., 2014, 2019, 2020). Furthermore, increases in central gain and plaque deposits in the IC are now being correlated to Alzheimer’s Disease and dementia (Na et al., 2023). However, it is extremely important to note that 1) age-related changes in the IC can be independent of cochlear deafferentation, central gain in aged listeners is not specific to pathology (e.g. tinnitus), and 2) features of hearing such as temporal responses to sinusoidal amplitude modulated stimuli and phase-locking to amplitude modulation are largely preserved with reduced GABA (Ouda et al., 2015; Johannesen and Lopez-Poveda, 2021; Burger and Pollak, 1998; Caspary et al., 2002; Bartlett et al., 2024).

As compromised neurotransmission in the IC can contribute to a range of neurological deficits, it is critical to better understand what changes in the IC are compensatory and at what point in time maladaptive changes predominate. Recent studies note that the middle-age brain has been understudied as compared to advanced ages, and because the biological mechanisms of aging are not a linear process interventions are often less effective as they are introduced too late (see review: Dohm-Hansen et al., 2024). As characteristics of age-related hearing loss are identifiable in a third of middle-aged adults and roughly 10 % of middle-aged individuals seek medical attention without clinical abnormality, it is increasingly important to assess multiple age groups (Arvin et al., 2013; Monini et al., 2015; Parthasarathy et al., 2020; Guo et al., 2024; Zink et al., 2024). One of the strengths of the current study is the evaluation of four age groups. Two of the groups, 19–20 months and 24 months represent different stages during middle age in our model, to demonstrate robust ultrastructural changes before, during, and after the characterized onset of hearing loss (Cai et al., 2018). Here we demonstrate many ultrastructural changes to GABAergic synapses between middle and old ages. Beside the density of GABAergic synapses, changes to terminal area, active zone length and vesicles at the active zone were closely aligned across the high and middle frequency; while changes in the low frequency were less robust (terminal loss and vesicles at the active zone) or occurred in an opposite fashion (terminal area). As stated above, we demonstrate changes to GABAergic neurotransmission during middle age, how these changes negatively or positively impact hearing remains to be determined. Ultimately, we do not know if any, or all, of our reported ultrastructural changes directly translate to specific functional changes to hearing. Many studies throughout the brain support the relationship that a loss of synapses results in a loss of function (see review Petralia et al., 2014). Furthermore, studies have demonstrated that the degree of synaptic loss correlates with the severity of the deficit (e.g. learning and memory; Smith et al., 2000). In the auditory system, at the very first synapse in the hair cells, it has been demonstrated that the loss of ribbon synapses/cochlear synaptopathy can have profound effects on the neural properties of the auditory nerve fibers (Kujawa and Liberman, 2009; Lin et al., 2011; Liberman, 2017). Taken together with the aforementioned central gain, we presume that the loss of GABAergic synapses in the aging IC will also contribute to alterations in neural processing. A finding from the current study that likely has direct links to functional parameters is the reduction of vesicles at the active zone during middle age. A logical functional outcome would be the release of less GABA, and therefore less overall inhibition of the postsynaptic target. It is curious though that we also found that active zones lengthen largely at old age. As mentioned above, the lengthening of synaptic active zones has been shown to provide as a compensatory mechanism indicating additional release of neurotransmitter (Levine et al., 1988; Helfert et al., 1999). Additional experiments will be needed to determine which ultrastructural changes have a direct functional outcome, and if these changes during old age are in response to potentially compensatory changes during middle age.

A major factor that may influence the balance of inhibition and excitation in the aging IC and explain how the IC might compensate for the reduction of GABAergic synapses is the upregulation of specific GABAA subunits during middle age (Milbrandt et al., 1997; Caspary et al., 1999; Robinson et al., 2019). GABA_A_ receptors, pentameric proteins that function as ligand gated chloride channels, mediate GABAergic inhibition in the IC (Smith, 1992; Palombi and Caspary, 1996). These receptors are essential to maintain temporal precision when acoustic stimuli are encoded (Caspary et al., 1999). The most common GABA_A_ subunit composition in the IC, and the mammalian brain, is α_1_ß_2_γ_2_ (Burt and Kamatchi, 1991; Wisden and Seeburg, 1992; Pirker et al., 2000). In the rat brain, the most common GABA_A_ subunit compositions include γ_2_ (α_1_ß_2_γ_2_, α_2_ß_2/3_γ_2_ and α_3_ß_n_γ_2/3;_ McKernan and Whiting, 1996; Caspary et al., 1999). However, with age, the composition of the GABA_A_ subunits shift such that the γ_1_ and α_2_ subunits are upregulated, and the maximal expression of each appears to be during middle-age (Milbrandt et al., 1997; Caspary et al., 1999; Robinson et al., 2019). This change in expression is presumably a compensatory response to the reduction of GABA release in the aging IC as these subunit compositions, GABA_A_R γ_1_ and GABA_A_Rα_2_ subunits, increase chloride permeability resulting in an elevated sensitivity to GABA (Wafford et al.,1993; Ducić et al., 1995; Milbrandt et al., 1997; Caspary et al., 1999; Dixon et al., 2017). It will be of great interest in our future studies to determine 1) whether surviving GABAergic synapses into old age are releasing GABA onto GABAA receptors with the γ_1_ and/or γ_2_ subunit, 2) whether γ_1_ and α_2_ subunits upregulation is specific to cell membrane location and how that may change from middle to old age, 3) if the age-related increase of perineuronal nets in the IC (Fader et al., 2016; Mafi et al., 2020) alter the plasticity for cells expressing specific GABAA subunits, and 4) what ICc cell types are expressing these upre-gulated subunits and whether we find nonuniform significant differences across the ICc axis as we have in the current study.

### 4.4. Conclusion

Overall, the current study demonstrates that GABAergic presynaptic terminals in the ICc undergo several robust ultrastructural changes and do so in a heterogenous manner across the dorsolateral to ventromedial axis. Although our animal model develops low frequency presbycusis, the greatest reduction of GABAergic synapses occurred in the high and middle frequency regions. It appears that most of the lost GABAergic input occurred on GABA-negative dendrites, potentially opening the door for increased excitability across the ICc. The use of four age groups highlighted that while changes to GABAergic ultrastructure began in early middle-age, significance was not routinely reached until late middle-age. Further investigations determining what GABAergic changes contribute to compensation and/or the onset of age-related hearing loss may help inform therapeutic approaches attempting to address the imbalance of excitation and inhibition that often leads to poorer temporal processing and speech encoding in much of the middle age and older adult population.

## Figures and Tables

**Table 1:** Summary of the ultrastructural characteristics of GABAergic synapses across the high frequency region of the ICc.

3–4 months	Area Examined (μm^2^)	# GABAergic synapses	Bouton Area (μm^2^)	Synaptic length (nm)	Ave. # mito	Mito grade	Vesicles (at synapse)	Vesicles (total)
B69 (R52) 3 mo	3200	100	0.73	240.04	1.3	4.4	9.23	68.1
B72 (R23) 3 mo	3200	105	0.64	203.6	1.1	4.3	8.8	59.6
B96 (R41) 3 mo	3200	140	0.8	223.9	1.3	4.6	7.15	56.9
B123 (R42) 4 mo	3200	86	0.78	247.2	1.9	4.3	7.7	70.7
B132 (R117) 3 mo	3200	103	0.78	240.5	1.4	4.4	8	73.8
**Totals/Averages**	**16,000**	**534**	**0.75**	**229.9**	**1.4**	**4.4**	**8.1**	**64.5**
19–20 months								
B78 (R63) 19 mo	3200	83	0.81	231.9	1.1	4.1	11.2	56.6
B81 (R98) 19 mo	3200	101	0.82	212.7	0.92	4.5	10.1	50.3
B84 (R99) 19 mo	3200	98	0.77	219.8	1.1	4.2	9.3	57.7
B105 (R84) 19 mo	3200	102	0.79	238.9	1.4	4.3	10.1	52
B141 (R96) 20 mo	3200	109	0.85	237.6	1.3	4.1	7.5	60.5
**Totals/Averages**	**16,000**	**493**	**0.81**	**228.3**	**1.2**	**4.2**	**9.5**	**55.2**
24 months								
B75 (R51) 24 mo	3200	82	0.95	243.5	1.89	3.75	8.1	66.6
B114 (R102) 24 mo	3200	83	0.84	237.1	1.75	4.2	7.8	67.4
B120 (R108) 24 mo	3200	76	0.99	216.6	2.1	3.8	9.5	77.3
B135 (R109) 24 mo	3200	79	0.85	218.1	1.8	4	5.3	58.6
B138 (R110) 24 mo	3200	79	0.85	229.3	0.98	3.8	5.3	36.6
**Totals/Averages**	**16,000**	**399** **	**0.86**	**229.2**	**1.7**	**3.9**	**7.2** ^	**61.7**
28–29 months								
B87 (R29) 28 mo	3200	68	0.99	264.1	1.6	3.6	5.8	72.7
B90 (R46) 28 mo	3200	88	0.92	256.3	1.4	3.5	5.7	51.8
B93 (R79) 29 mo	3200	75	0.68	288.5	1.5	3.5	4.6	35.5
B99 (R45) 28 mo	3200	72	0.83	257	1.7	3.7	7.01	66.3
B126 (R47) 29 mo	3200	77	0.69	234.4	1.3	3.9	6.6	57.7
**Totals/Averages**	**16,000**	**380** ** ^	**0.82**	**259.8** ** ^ #	**1.5**	**3.6** ** ^	**5.9** * ^	**56.4**

* p < 0.05 as compared to 3–4 months

** p < 0.01 as compared to 3–4 months

^ p < 0.05 as compared to 19–20 months

# p < 0.05 as compared to 24 months

**Table 2:** Summary of the ultrastructural characteristics of GABAergic synapses across the middle frequency region of the ICc.

3–4 months	Area Examined (μm^2^)	# GABAergic synapses	Bouton Area (μm^2^)	Synaptic length (nm)	Ave. # mito	Mito grade	Vesicles (at synapse)	Vesicles (total)
B70 (R52) 3 mo	3200	97	0.7	235.3	1	4.3	7.9	36.6
B73 (R23) 3 mo	3200	102	0.79	224.2	1.9	3.99	10.9	70.8
B97 (R41) 3 mo	3200	143	0.79	223.6	1.8	4.12	5.9	51.4
B124 (R42) 4 mo	3200	89	0.78	238	2.2	4.7	8.3	65.1
B133 (R117) 3 mo	3200	113	0.63	243	1.1	4.4	11.2	56.7
**Totals/Averages**	**16,000**	**544**	**0.74**	**232.2**	**1.6**	**4.3**	**8.7**	**55.6**
19–20 months								
B79 (R63) 19 mo	3200	102	0.81	225.4	1.1	4.2	6.7	78.1
B82 (R98) 19 mo	3200	95	0.84	236.5	1.3	4.1	7.3	60.2
B85 (R99) 19 mo	3200	97	0.8	235	1.1	4.3	6.6	63
B106 (R84) 19 mo	3200	96	0.81	225.3	1.2	4.3	7.2	47.1
B142 (R96) 20 mo	3200	121	0.8	230.1	1	4.2	8.3	55.1
**Totals/Averages**	**16,000**	**511**	**0.81**	**230.4**	**1.1**	**4.2**	**7.3**	**60.1**
24 months								
B115 (R102)24 mo	3200	93	0.84	235.4	1.5	3.9	8.4	60.8
B118 (R100)24 mo	3200	80	0.9	234.9	0.95	4.1	5.4	38
B121 (R108) 24 mo	3200	60	0.88	254.3	1.78	3.7	5.9	59.5
B136 (R109) 24 mo	3200	74	0.83	222.9	1.1	3.8	8.3	44.2
B139 (R110)24 mo	3200	86	1.02	240.8	1.87	3.8	9.7	61.5
**Totals/Averages**	**16,000**	**393** ** ^	**0.9** *	**236.8**	**1.5**	**3.8**	**7.7**	**53.3**

* p < 0.05 as compared to 3–4 months

** p < 0.01 as compared to 3–4 months

^ p < 0.05 as compared to 19–20 months

# p < 0.05 as compared to 24 months

**Table 3:** Summary of the ultrastructural characteristics of GABAergic synapses across the low frequency region of the ICc.

3–4 months	Area Examined (μm^2^)	# GABAergic synapses	Bouton Area (μm^2^)	Synaptic length (nm)	Ave. # mito	Mito grade	Vesicles (at synapse)	Vesicles (total)
B71 (R52) 3 mo	3200	105	0.73	226.6	1.2	4.2	7.8	41.3
B74 (R23) 3 mo	3200	116	0.76	222.6	1.6	4.5	8.85	61.1
B98 (R41) 3 mo	3200	95	0.83	218.8	2.1	4.6	3.9	69.5
B125 (R42) 4 mo	3200	115	0.77	252.2	1.9	4.2	7.5	76.6
B134 (R117) 3 mo	3200	117	0.71	249.5	1.1	4.3	10.8	65.7
**Totals/Averages**	**16,000**	**548**	**0.76**	**234.7**	**1.5**	**4.4**	**7.9**	**62.2**
19–20 months								
B80 (R63) 19 mo	3200	92	0.89	244.1	1.3	4.4	6.5	55.7
B83 (R98) 19 mo	3200	119	0.86	237.8	1.3	4.3	6.4	42.3
B86 (R99) 19 mo	3200	99	0.85	236.1	1.01	4.2	8.6	57.7
B107 (R84) 19 mo	3200	124	0.86	227.2	1.1	4.2	7.8	54.2
B143 (R96) 20 mo	3200	101	0.81	242.2	1.2	4.3	9.2	64.2
**Totals/Averages**	**16,000**	**535**	**0.86**	**237**	**1.2**	**4.2**	**7.7**	**54.4**
24 months								
B113 (R101) 24 mo	3200	83	0.87	269	0.92	3.7	5.4	38.7
B116 (R102) 24 mo	3200	103	0.84	238.9	1.5	3.6	7.9	57.8
B122 (R108) 24 mo	3200	95	0.82	242.8	1.7	3.5	5.7	58.9
B137 (R109) 24 mo	3200	83	0.78	261.6	1.3	3.6	7.8	60.5
B140 (R110) 24mo	3200	76	0.81	255.1	1.2	3.8	6.1	40.5
**Totals/Averages**	**16,000**	**440**	**0.83**	**252.5**	**1.3**	**3.6** ** ^	**6.6**	**51.1**
28–29 months								
B89 (R29) 28 mo	3200	85	0.75	235.9	1.1	3.7	5.6	68.4
B92 (R46) 28 mo	3200	77	0.89	223.8	1.5	4	6.9	80.3
B95 (R79) 29 mo	3200	93	0.61	245.5	1.3	3.6	5.3	44.6
B101 (R45) 28 mo	3200	81	0.77	248.2	1.3	3.7	7.6	65.3
B128 (R47) 29 mo	3200	86	0.75	249.1	1.9	3.5	7.3	65.1
**Totals/Averages**	**16,000**	**422**	**0.75** ^	**238.5**	**1.4**	**3.7** ** ^	**6.3**	**63.01**

* p < 0.05 as compared to 3–4 months

** p < 0.01 as compared to 3–4 months

^ p < 0.05 as compared to 19–20 months

# p < 0.05 as compared to 24 months

**Table 4:** Summary of correlation coefficent p-values and spearman’s rho values collapsed across ages.

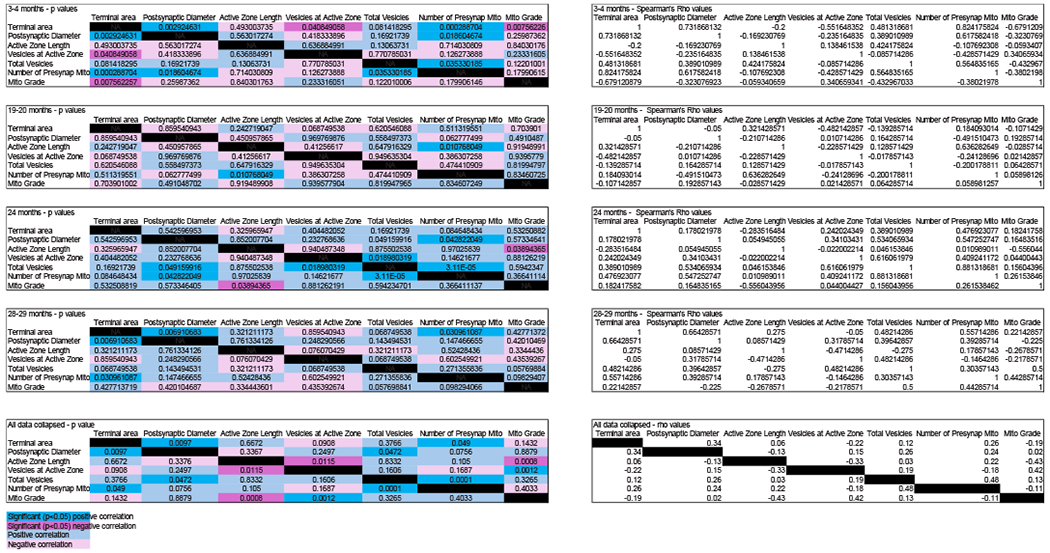

## Abbreviations:

FBN: Fischer Brown Norway
GABA: gamma-Aminobutyric acid
GAD: glutamic acid decarboxylase
IC: inferior colliculus
ICc: central nucleus of the inferior colliculus
ICd: dorsal cortex of the inferior colliculus
IClc: lateral cortex of the inferior colliculus
LD: large dendrite
MD: medium dendrite
Pre G-: GABA positive presynaptic terminal
Pre G-: GABA negative presynaptic terminal
SD: small dendrite
